# Multiplexed longitudinal analysis of the cellular and microbial dynamics of acute polymicrobial sepsis in mice

**DOI:** 10.3389/fimmu.2025.1682451

**Published:** 2025-12-03

**Authors:** Tori E. Peacock, Kenny Johnson, Abhinav R. Cheedipudi, Ahmed D. Mohammed, Ryan A. W. Ball, J. Hunter Cox, Savannah K. Pender, Amy Jolly, Kandy T. Velázquez, Jay Potts, Angela Murphy, Norma Frizzell, Colin Evans, Jason L. Kubinak

**Affiliations:** 1 Department of Pathology, Microbiology, and Immunology, School of Medicine, University of South Carolina, Columbia, SC, United States; 2 Department of Pharmacology, Physiology, and Neuroscience, School of Medicine, University of South Carolina, Columbia, SC, United States; 3 Department of Cell Biology and Anatomy, School of Medicine, University of South Carolina, Columbia, SC, United States

**Keywords:** sepsis, blood biome, septic shock, spectral flow cytometry, emergency myelopoiesis

## Abstract

**Introduction:**

Acute polymicrobial sepsis is a life-threatening emergency caused by the body’s immune response to bloodstream infection by two or more microbes. Early detection and management of sepsis have been the focus of global survey programs, driven by its association with hospital readmissions and long-term adverse health outcomes.

**Methods:**

Animal models are essential tools for studying mechanisms of sepsis pathogenesis and the only way to empirically dissect the acute phase of disease. With this in mind, the goal of the current study was two-fold: to demonstrate the feasibility of performing multiplexed longitudinal assessment of acute sepsis pathogenesis and to emphasize the granularity with which acute sepsis can be studied using this method. Using the fecal suspension test (FST) model of acute polymicrobial sepsis in C57BL/6 mice we simultaneously characterize hematological, immunological, and microbiological aspects of acute sepsis induction.

**Results:**

Our data shows that high dimensional flow cytometry paired with flow-based plasma cytokine measurements captures the dynamic shift from pro-inflammatory to anti-inflammatory immune responses during an acute septic event; highlighting the role of emergency myelopoiesis in this process. Additionally, myeloid cell heterogeneity is characterized and strongly implicates the emergence of myeloid derived suppressor like cells (MDSC-like cells) as central to this switch. Furthermore, we demonstrate a 16S-based method for studying the blood biome that allows for discrimination between endogenous (bacterial DNAemia) and exogenous (actively growing bacteria in blood) sources of microbial DNA. Using this approach, we demonstrate that polymicrobial sepsis in our model is due to outgrowth of *Enterococcus* and *Staphylococcus*; two genera of bacterial pathobionts commonly observed in human sepsis patients. Finally, using several assessments of disease severity, we demonstrate stratification of septic mice into survivors and non-survivors and show how pre-septic immune assessment can be used to identify potential biomarkers of sepsis risk.

**Discussion:**

Collectively, the approach we describe simultaneously reduces research animal use, strengthens scientific rigor, provides a pre-clinical platform for biomarker discovery and the study of therapeutic interventions, and most importantly advances our ability to study the acute phase of sepsis that carries a high mortality rate and is difficult to prospectively study in humans.

## Introduction

Sepsis is a leading cause of death in humans, accounting for 19.7% of all reported global deaths in a 2019 survey (1–7). Sepsis can rapidly induce death due to the development of hypoxia within the tissues of vital organs (e.g. heart, brain, kidneys, liver) leading to organ failure. Sepsis-induced hypoxia can be driven by acute respiratory failure, lowered blood pressure (septic shock), or coagulation within the microvasculature (disseminated intravascular coagulation (DIC)). Reported mortality rates for sepsis vary widely from 17.5% to 46.2% (8–10) and associated mortality is increased upon development of secondary complications. A tremendous body of work has been done to understand mortality risk in chronically ill septic patients, where longitudinal analyses can be readily performed. However, less progress has been made in our understanding of the mechanisms and risk factors driving acute sepsis mortality. Here, animal models (with due acknowledgement of all their limitations (11, 12)) become an essential tool to study pathogenesis.

Our current understanding of the immunology of sepsis revolves around the notion that sepsis causes immune dysregulation characterized by a sequential shift from an initially hyperinflammatory state to an immunosuppressive one (13, 14). Functionally, the hyperinflammatory response is characterized by an initial wave of proinflammatory cytokines (e.g. IL-6, TNFα, IL-1β, etc.)(cytokine storm) produced by innate immune cells activated in response to microbial signals and signals derived from damaged tissues. This phase is followed by a secondary wave of anti-inflammatory cytokines (e.g. IL-10, TGFβ, etc.) resulting in chronic immunosuppression (termed ‘immune paralysis’) (15). In patients that recover from sepsis, the development of chronic immune paralysis is associated with secondary complications and increased mortality risk (16).

Myeloid cells are the primary instigators of the immune response during an acute septic event. A current focus of sepsis research is on understanding how sepsis influences myeloid cell heterogeneity. In both humans and mice, sepsis has been shown to impact myelopoiesis (17), with effects that can persist long after the septic event has ended. Sepsis induces emergency hematopoiesis (i.e., the rapid development of immune cells in the bone marrow in response to infection) and has been shown to skew hematopoietic stem cell (HSC) differentiation toward the production of myeloid-derived suppressor cells (MDSCs) (18, 19). MDSCs have been shown to be enriched in septic patients and associated with poorer long-term clinical outcomes (18, 20). Therefore, assessing MDSCs, alongside other immune cell populations and cytokines, is critical for a holistic understanding of how the immune landscape shifts in response to sepsis and will provide deeper insights into underlying mechanisms leading to immune paralysis.

The dissemination of microbes from the gut into the abdomen (intra-abdominal sepsis) is a common cause of sepsis in patients and has a particularly poor clinical outcome (21–23). Intra-abdominal sepsis oftentimes results from a polymicrobial infection (the presence of two or more microbes) within the systemic compartment (24, 25). Due to the lack of resolution regarding the ecology of sepsis, it has been argued that the incidence of polymicrobial sepsis is likely under-reported (26). However, we do know that chronic sepsis patients have altered lung (27) and gut (28, 29) microbiomes indicating that the physiological state induced by sepsis can alter microbial ecology in distal sites and/or that certain microbiomes may be more prone to dissemination within the systemic compartment. Whether and how these ecological shifts influence sepsis mortality are unknown. Equally unclear is how the dynamics of polymicrobial colonization of the bloodstream during a septic event influences the ensuing immune response and consequently disease severity. This is due in large part to the lack of development of methodologies to study the dynamics of polymicrobial colonization of the bloodstream.

Longitudinal analysis of the cellular and microbial dynamics of an acute life-threatening polymicrobial septic event is not possible to study in humans and is a critical gap in our understanding of sepsis pathogenesis. Given this, laboratory mice become an indispensable tool for mechanistically dissecting the initial stages of sepsis-induced immune dysregulation. Here, we describe an elaboration to the established fecal suspension test (FST) model of polymicrobial sepsis that allows for longitudinal assessment of hematological, immunological, and blood microbiome parameters (from the same animals) during the critical 21hrs after an acute septic event. We demonstrate the capacity of our approach to capture the switch between hyperinflammation and immune suppression, to characterize the effect of emergency hematopoiesis on myeloid cell heterogeneity, and to track the dynamics of microbial colonization of the bloodstream. The relevance of our approach to the study of human sepsis is discussed.

## Methods

### Experimental animals

Thirty 8-week-old WT C57BL/6 mice were purchased from Jackson Laboratories [CAT#000664: 15 males (M) and 15 females (F)] for use in the outlined experiments. Animals were reared and maintained under identical SPF conditions in a single environmentally-controlled room. Animals were maintained under constant environmental conditions (70°F, 50% relative humidity, 12:12 light:dark cycles) and given *ad libitum* access to autoclaved drinking water and a standard mouse chow. Sex as a biological variable was considered in our model with no statistically significant differences observed between male and female mice in hematological, immunological, or disease severity readouts either before, or after, sepsis induction (**Supplementary Figure S1**). All animal use strictly adhered to federal regulations and guidelines set forth by the University of South Carolina Institutional Animal Care and Use Committee (Protocol#101882).

### Blood collection

**Figure 1:**
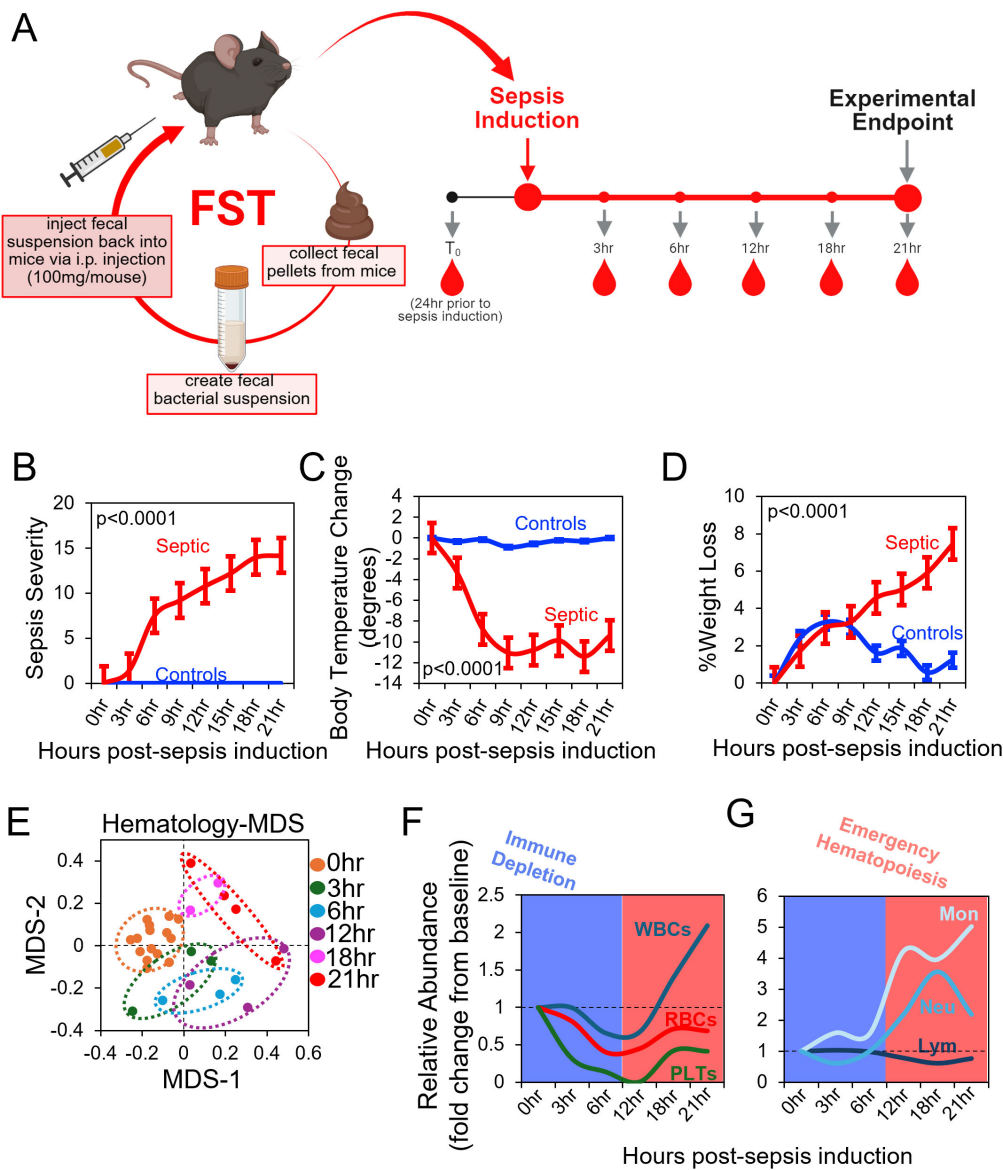
A longitudinal FST model to study the immune-microbial dynamics of polymicrobial sepsis. **(A)** A schematic of the experimental design is shown. **(B)** Sepsis-induced sickness behavior was estimated using a seven-point scoring system. **(C)** Weight loss was calculated as the percentage of initial weight lost in response to sepsis induction. **(D)** The change in body temperature (from baseline) was calculated for septic mice. **(E)** An MDS plot is shown to demonstrate the hematological dynamics of polymicrobial sepsis based on twenty blood phenotyping parameters. **(F, G)** Shifts in the relative abundance of major blood cell **(F)** and immune cell **(G)** subsets (fold change from baseline) over the time-course of the experiment are shown. **(B-D)** p-values represent results of Students t-test based on comparison of endpoint (21hr) measures.

Blood was collected twice from each mouse; once prior to sepsis induction [initial timepoint=T_0_ (**Figure 1A**)] and once after euthanasia (endpoint). Whole blood (50-75μL) was drawn via tail snip from all mice 24hrs prior to sepsis induction using heparinized capillary tubes. Hematological assessment and immunophenotyping was performed on these samples to obtain initial timepoint data. At experimental endpoints, mice were euthanized via CO_2_ and whole blood was collected via cardiac puncture. For time-series measurements, three mice (of mixed sexes) were euthanized at defined timepoints and blood was collected via cardiac puncture for downstream analysis: 3hr (2F,1M), 6hr (1F,2M), 12hr (2F,1M), 18hr (1F,2M), and 21hr (5F, 4M).

### High-dimensional spectral flow cytometry

A 28-marker flow cytometry panel was designed to broadly characterize blood immune-phenotypes of experimental mice (see **Supplementary Table T1** for a list of markers, concentrations used, and vendor information). *Cell isolation:* 2mLs of RBC lysis buffer (Thermofisher) was added to 25μl-50μl of whole blood and allowed to incubate at room temperature for ten minutes. The sample was then centrifuged at 2500 revolutions per minute (RPM) for ten minutes. The supernatant was discarded, and samples were resuspended in 300μL of complete RPMI (RPMI 1640 supplemented with FBS, sodium pyruvate, non-essential amino acids, L-glutamine, penicillin-streptomycin, and β-ME). Cell suspensions were plated in a 96 well plate and centrifuged at 1350 RPM for five minutes. *Cell staining:* Cells then resuspended in 100μL of Fc-receptor block (Biolegend) and incubated on ice for ten minutes. The plate was then centrifuged at 1350 RPM, the supernatant was discarded, and cells were suspended in 100μL of column buffer (1X HBSS, 5% FBS, and 0.5 M EDTA) containing the antibody cocktail. Cells were stained at 4°C for twenty minutes. The plate was then centrifuged at 1350 RPM for five minutes, the supernatant was discarded, and cells were resuspended in 100μL of 1x PBS containing 1:500 Zombie-Aqua viability dye. Cells were stained at room temperature and protected from light for fifteen minutes. The plate was then centrifuged at 1350 RPM for five minutes and the supernatant was discarded. Cells were then washed three times by resuspension in 250μL of column buffer, gentle agitation by pipetting, and centrifugation at 1350 RPM for five minutes. After the third wash, cells were fixed in 500μL of 2% paraformaldehyde*. Data collection and handling:* Flow cytometry analysis was performed using a FACSDiscover S8 Imaging Spectral Cell Sorter. SpectraComp beads (Slingshot Biosciences) were used to set detector gains during initial experimental template setup on the instrument. Data collection involved pre-gates to eliminate dead cells (Zombie-Aqua^hi^) and debris/doublets, and to minimize contaminating RBC events (Ter119^hi^)(**Supplementary Figure S2**). For each sample, 200,000 events defined as single cells, live, and TER119^lo/-^ were recorded. Supervised (i.e. manual) gating to define 14 cellular subsets was performed using FlowJo 10 Software (BD Biosciences). Events were divided by CD45 positivity into immune cells and non-immune cells. Non-immune cells were further refined to platelets by CD61 and CD41 positivity. CD45^+^ and CD45^-^ cell fractions were concatenated separately with equal sampling from each sample and gated manually (**Supplementary Figures S3–5**)(**Supplementary Table T2**).

### Histology

To assess DIC phenotypes, vital organs (kidneys) were collected from euthanized mice, fixed in 10% formalin, and then stored in 70% ethanol until processing. Paraffin-embedded kidney sections (longitudinal cuts to maximize visualization of glomeruli in the renal cortex) were mounted and stained using the Martius scarlet Blue stain (VitroView) for visualization of thrombi (**Supplementary Figure S6**). Image J Macro programming language was used to automate the glomeruli selection and fibrin clot area from the 10x Revolve Kidney Cortex images. The Bowman’s Capsule was used as the landmark for glomeruli selection. The specific region selection coordinates were saved using the ROI manager tool. For each image, the respective region from the ROI manager was placed on the image, everything outside of the polygon region was cleared, Image J’s color deconvolution’s H&E2 splitting technique was placed on the image, and a new image was generated using the operation channel three difference channel two. Then the new processed image was thresholded and converted to mask using red thresholding in the regions (125, 255, raw), and the measure operator was used to gather the area of the fibrin clot (percent area) and the area of the glomeruli within the respective ROI region. A minimum of sixty six glomeruli were analyzed per mouse.

### Fecal suspension test

We followed a previously established protocol for performing the fecal suspension test (30). Briefly, fresh fecal pellets were collected from all experimental mice on the morning of the day of sepsis induction, pooled, and then homogenized in sterile 1X phosphate-buffered saline. Course materials were precipitated by spinning the fecal slurry at 12,000 RPM for ten minutes and then supernatants were filtering through a 40μm cell strainer. The fecal suspension was diluted to 500mg/mL in sterile 1X phosphate-buffered saline and sepsis was induced by performing intraperitoneal (i.p.) injection to introduce 200µL of inoculum (standardized 100mg/mL dose per mouse) into the body cavity.

### Physiological readouts

Body temperature of each mouse was measured immediately prior to sepsis induction and then every three hours following induction (3hr, 6hr, 9hr, 12hr, 15hr, 18hr, and 21hr). Body temperature was measured using an infrared laser temperature gun on the abdomen. At the same time, body weight was also measured immediately prior to sepsis induction and then every three hours following induction (3hr, 6hr, 9hr, 12hr, 15hr, 18hr, and 21hr).

### Behavioral assessment

A previously defined seven-point scoring rubric was used to assess the severity of sepsis in mice (31). Sepsis severity was scored at the initial timepoint (immediately prior to sepsis induction), and then at 3hr, 6hr, 9hr, 12hr, 15hr, 18hr, and 21hr post-sepsis induction. In brief, the scoring rubric considers eye deficits, response to stimuli, locomotion, activity, appearance, respiration rate, and respiration quality on a scale of 0-4. The cumulative score indicates sepsis severity, with a higher score indicating more severe illness. Cumulative sepsis severity scores are a combination of the scoring of eye deficits, response to stimuli, locomotion, activity, appearance, respiration rate, and respiration quality. Each is scored 0–4 and these are then added together to calculate the cumulative sepsis severity score.

### Hematological assessment

50-75μL of whole blood was collected in heparinized capillary tubes for hematological assessment and flow cytometry. Whole blood (25μL) was placed in an Eppendorf tube and twenty hematological parameters were simultaneously measured using a VetScan HM5 instrument.

### Multiplex plasma cytokine analysis

At experimental endpoints, whole blood (approximately 500uL) was placed in EDTA coated 1.5mL microfuge tubes and plasma was separated by centrifugation. Plasma was stored at -80°C until use. Plasma samples were thawed once and immediately used. Thirteen plasma cytokines were simultaneously measured via flow cytometry using the Legendplex Cytokine Release Syndrome Kit (Biolegend). Kit-specific software (Qognit) was used to calculate cytokine concentrations. Cytokine data was collected using a BD FACSymphony A5 cell analyzer. All samples were run on a single plate and values below the level of detection for the kit (defined in kit protocol) were excluded from analysis. No compensation is involved as PE is the only fluorophore used in this kit. Instrument setup and data analysis steps are described in the kit protocol.

### 16S rRNA gene sequencing

Approximately 500μL of whole blood was collected from each mouse at defined timepoints, placed in ETA coated 1.5mL microfuge tubes and frozen at -80°C until use. DNA was isolated from mouse blood using the QIAamp UCP Pathogen Mini Kit (Qiagen) with bead-beating Pathogen Lysis Tubes (Qiagen). To characterize the fecal microbiome representing our sepsis inoculum, DNA was isolated from our fecal slurry using the QiaAMP Powerfecal DNA Extraction Kit (Qiagen) with bead beating. Isolated DNA was sent to the University of Alabama Heflin Genomics Core for 16S rRNA gene sequencing using standard primers (F515-R806) targeting the V3-V4 hypervariable region. Raw fastq files were processed using QIIME2.0 software (32). A Q-score cutoff of >20 was used. Unassigned reads and singletons were filtered out prior to analysis. QIIME2.0 software was then used for analysis of filtered OTU tables (**Supplementary Table T3**). For consideration of the blood biome, the contribution of environmental contaminants (in buffer (PBS) and anti-coagulant (EDTA) used to collect blood) and bacterial DNAemia was corrected for by subtracting OTU sequence reads obtained from these control samples from the respective sepsis mouse OTUs. For DNAemia, the composition of 16S reads derived from control mice was used to establish baseline OTU correction values. Using this correction method, only OTUs that were enriched in (or unique to) septic mice (i.e. above background DNAemia and not present as contaminants in reagents) were considered in our blood biome analysis.

### Plasma lactate dehydrogenase measurement

At experimental endpoints, whole blood (~500μL) was placed in a heparinized 1.5mL microfuge tube and spun at 14,000 RPM for ten minutes to separate plasma. Plasma was placed in a new 1.5mL microfuge tube and stored at -80°C until use in downstream assays. Plasma lactate levels were measured using a Lactate Dehydrogenase (LDH) Colorimetric Activity Kit following kit instructions (ThermoFisher).

### Mass spectrometry quantification of succination in plasma

The quantification of S-2-succinocysteine (2SC) was performed as we have previously described, with minor modifications (33). Plasma (50μL) was precipitated with 200μL methanol (ThermoFisher Scientific) containing 10 pmol (13)C_3_ (15), N 2-succinocysteine (2SC) per sample as an internal standard. The samples were incubated on ice for twenty minutes before centrifuging at 13,000 x g for ten minutes at 4°C. The supernatant containing polar metabolites was dried *in vacuo*, before resuspension in 1mL 0.1% trifluoroacetic acid (TFA, Acros Organics). Solid Phase Extraction (SPE) was performed using 1mL C18 SPE cartridges (Waters) with 0.1% TFA/40% methanol as the eluent. The samples were dried *in vacuo* prior to derivatization with 100:20 v/v ice-cold ethanol/acetyl chloride (Mallinckrodt Chemicals) at 55°C for 2 h before drying *in vacuo*, The reaction products were resuspended in 200µL (20:80 v/v) acetonitrile/water and transferred to autosampler vials for LC-MS/MS analysis. LC separation was performed on a Thermo Vanquish Flex liquid chromatography system using a Waters XBridge C18 Reverse phase column (Waters Corp.). The column was 2.1 mm by 100mm with 3.5µm particles. The column was maintained at 40°C. Following a 3µl sample injection, a binary gradient elution was performed. Solvent A consisted of water with 0.1% formic acid and solvent B contained acetonitrile with 0.1% formic acid. The separation began with the column equilibrated at 10% B for 1.5 min. then ramped to 95%B at 10 min and held there until 15 min. The system then returned to 10% B for 8 min. The column flow rate was 0.2µl/min. Positive ion electrospray mass spectra were acquired on a Thermo Q-Exactive HF-X Quadrupole-Orbitrap performing parallel reaction monitoring (PRM). Precursor ions (322, 326 Da) were isolated by the quadrupoles using a 0.5 m/z isolation window and fragmented in the higher-energy collisional dissociation (HCD) cell using stepped collision energy (CE) (15,25,35 eV); the automatic gain control (AGC) target was 1E6 and maximum ion trap (IT) was set at 150 ms. The Orbitrap resolution used for PRM was 30,000. The source capillary temperature was 275°C, other MS source settings were sheath gas flow: 45; auxiliary gas flow: 10; sweep gas flow: 2; spray voltage: 3.5 kV; funnel RF level: 40; auxiliary gas temperature: 400°C. XCalibur™ 4.2 software (Thermo) was used to construct extracted ion chromatograms of the transitions 322→205 (2SC), and 326→205 (13)C_3_ (15), N-2SC). The area of the 2SC peak was normalized to the area of the (13)C_3_ (15), N 2SC internal standard to obtain peak area ratios. The mass of 2SC in the plasma samples was normalized to the plasma volume to calculate free 2SC concentrations.

### Statistics

**Figure 2:**
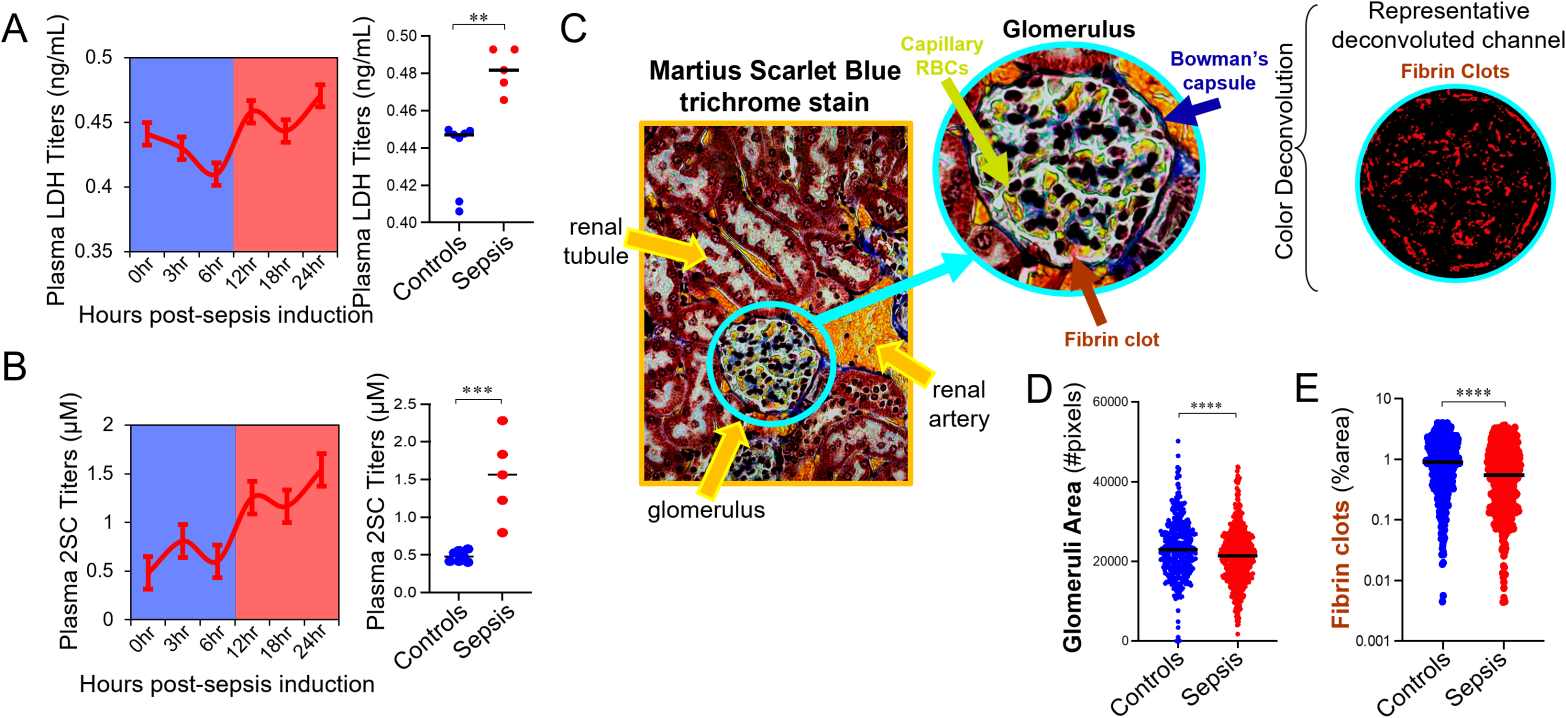
Hypoxia induced by FST model is caused by shock and not DIC. **(A)** Endpoint plasma LDH concentrations to assess hypoxia are compared between control and septic mice. Mann-Whitney U test, **=p<0.01. **(B)** Endpoint plasma 2SC concentrations are compared between control and septic mice. Student’s t-test; ***=p<0.001. **(C)** Representative Martius scarlet blue (MSB) trichrome staining of glomeruli within the renal cortex of a mouse is shown. Glomeruli area was calculated as the number of total image pixels. Color deconvolution was used to quantify the percentage of glomerulus area containing red (fibrin clots) or yellow (capillary RBC content) pixels. **(D, E)** Glomeruli area **(D)** and fibrin clot content **(E)** are compared between control and septic mice 24hrs post sepsis-induction. A minimum of 66 glomeruli were analyzed per mouse and the data in C and D represents individual glomeruli measurements made across n=5 mice per treatment group. Dunn’s test; ns=not significant, ***=p<0.001, ****=p<0.0001.

Prism10.0 (GraphPad) was used for univariate pairwise statistical comparisons. Normality was assessed using the Shapiro-Wilk’s test. For normally-distributed datasets, means were used to indicate our measure of central tendency with pairwise t-tests (two groups) or multiple t-tests (three or more groups) performed. For non-normally-distributed datasets, medians were used to indicate our measure of central tendency with Mann-Whitney (two-comparison) or Kruskall-Wallis tests (three or more groups) performed. Where appropriate, statistical (false discovery rate) corrections were applied to account for multiple hypothesis testing. All multivariate statistics were performed using JMP16 Software (SAS). To minimize the effect of non-normally distributed datasets on multivariate analyses, all datasets were log-transformed prior to multivariate analysis. Calculation of immune dysregulation was performed as previously described (34). Multidimensional scaling (MDS) plots were generated to visually represent relationships among experimental groups. All multivariate plots shown in figures were generated in Microsoft Excel. Of note, all time-series patterns should be considered qualitative in nature. Because of U.S. Federal limitations to the amount of blood that can be collected from the same mouse in a 24hr period, it was not possible to perform longitudinal blood collections from the same animals. Thus, we could not perform repeated measures ANOVA on time-series datasets. We instead provide end-point comparisons for all time-series patterns. All data points shown in figures reflect experimental sample size, with the exception of **Figure 2E** which reflects bi-directional distance measures between data-points”.

## Results

### The FST mouse model of acute sepsis

To study acute septic events, we utilized the FST model of sepsis (30). Our FST model involves the collection of fecal pellets from experimental mice immediately prior to sepsis induction to generate a pool of fecal bacteria for downstream intraperitoneal (i.p.) injection in mice (**Figure 1A**). Dose was standardized by fecal weight, with each mouse receiving fecal bacteria proportional to 100mg. Mice were i.p. injected with fecal bacteria and monitored hourly for the duration of the study with subsets of animals sacrificed at defined timepoints (3hr (n=3), 6hr (n=3), 12hr (n=3), 18hr (n=3), and 21hr (n=9)) for sample collection (**Figure 1A**). In parallel, control mice were sham injected with PBS vehicle. FST-induced sepsis followed the expected phenotypes associated with other sepsis models. Specifically, septic mice exhibited increased sepsis severity scores (**Figure 1B**), hypothermia (**Figure 1C**), and weight loss (**Figure 1D**). Time-dependent abnormalities in twenty hematological measurements were noted during the sepsis time-course (**Figure 1E**). The most notable time-dependent shifts in hematological values involve reductions in blood cell populations (WBC, RBCs, and platelets) within the first twelve hours of sepsis (**Figure 1F**), which we hereafter term the “immune depletion” phase of acute sepsis. Importantly, the observed decrease in WBC abundance is attributable to lymphopenia (**Supplementary Figure S7**), a commonly observed phenotype in severe sepsis and has been tied to worse clinical outcomes (39304908). Following this initial loss of blood cells, RBC and platelet abundance rebounded to normal levels while immune cell abundance dramatically increased (**Figure 1F**). WBC expansion was primarily driven by increasing abundance of myeloid cells in circulation, a characteristic feature of infection-driven emergency myelopoiesis (35, 36) (**Figure 1G**).

Plasma lactate dehydrogenase (LDH) levels were quantified to measure the extent of tissue hypoxia induced by acute polymicrobial sepsis (indirect measure of septic shock), with plasma LDH levels dropping during the immune depletion phase and then significantly rising during the emergency hematopoiesis phase of the septic response (**Figure 2A**). Krebs cycle intermediates in plasma have been shown to be predictive of sepsis mortality in humans (37). Increased levels of fumarate and the subsequent succination of protein cysteines to generate S-2-succinocysteine (2SC) is associated with the immunometabolic response to lipopolysaccharide in macrophages (38), but protein succination has not been documented in models of endotoxemia or sepsis. Mitochondrial metabolic remodeling occurs in response to an immune stimulus, and mitochondrial stress and pseudohypoxia are known to increase protein succination (33, 39). Consistent with these observations, mass spectrometry analysis demonstrated a significant increase in plasma free S-(2-succino) cysteine (2SC) levels in septic versus control mice (**Figure 2B**). Given the platelet consumption observed during the immune depletion phase, we wanted to assess whether disseminated intravascular coagulation was a contributing factor to the elevated hypoxia in septic mice. MSB trichrome staining of kidneys (**Figure 2C**) demonstrated that 21hrs post-sepsis induction kidney glomeruli were smaller (**Figure 2D**) with fewer fibrin clots (**Figure 2E**), which is inconsistent with DIC. Thus, hypoxia induced by the FST model of acute sepsis is driven by a shock response and not DIC.

### Sepsis dynamically alters the composition of circulating myeloid cells

**Figure 3:**
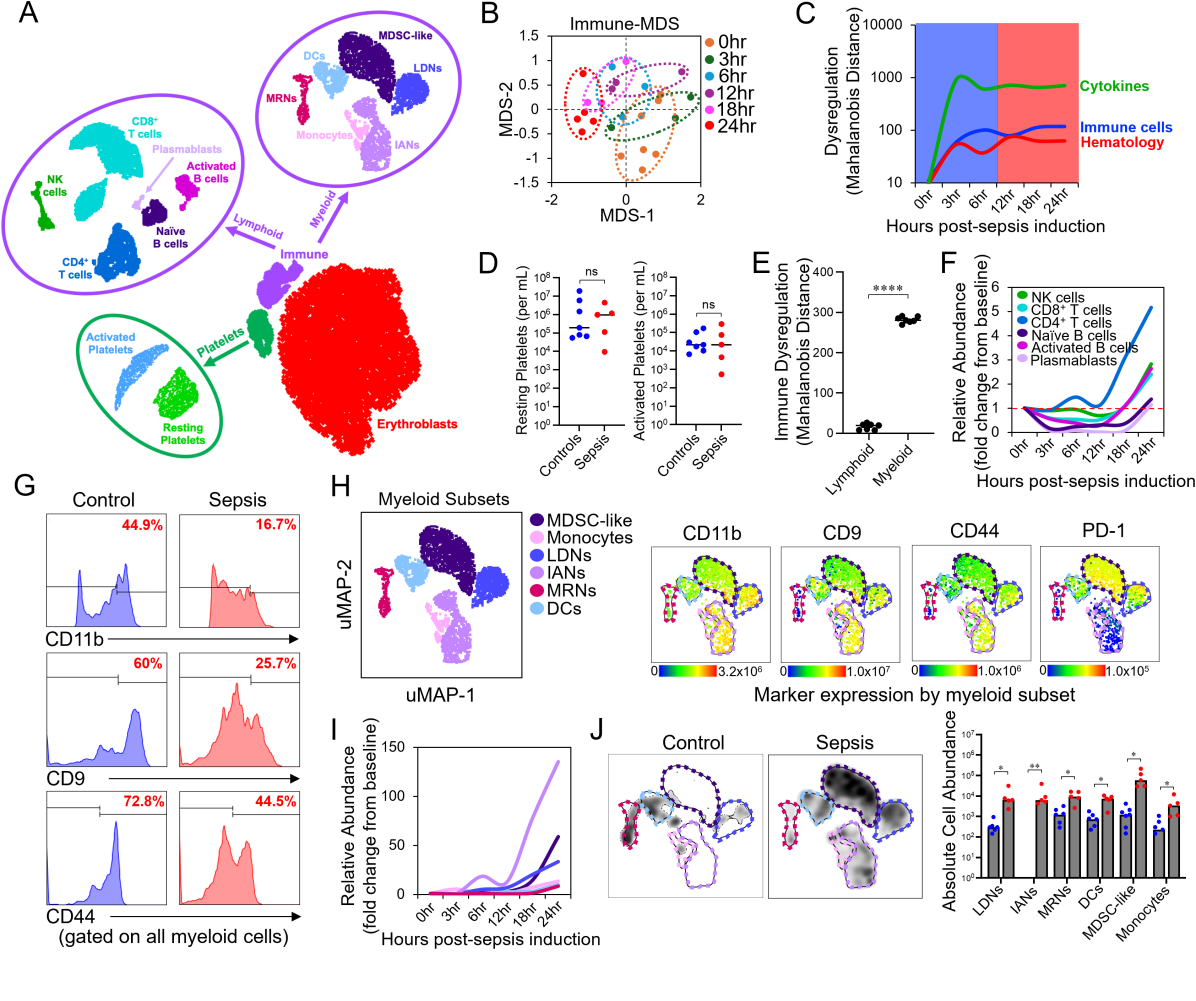
Polymicrobial septic shock dynamically alters the composition of circulating myeloid cells. **(A)** Representative schematic of blood cell subsets analyzed by high dimensional flow cytometry. **(B)** An MDS plot is shown to demonstrate the shift in immune status over time in response to polymicrobial sepsis. **(C)** The trend in the magnitude of dysregulation in hematological, immune, and cytokine responses to sepsis are shown over time. **(D)** The absolute abundance of resting and activated platelets at the experimental endpoint is compared between control and septic mice. Mann-Whitney U test; ns=not significant (p>0.05). **(E)** The contribution of shifts in abundance of lymphoid and myeloid cells to immune dysregulation in septic mice is shown. Student’s t-test; ****=p<0.0001. **(F)** Shifts in the relative abundance of lymphoid cell subsets (fold change from baseline) over the time-course of the experiment are shown. **(G)** Representative histograms are shown comparing the expression of adhesionmolecules on myeloid cells between control and septic mice. **(H)** A representative uMAP of analyzed myeloid cell subsets is shown along with heatmaps depicting expression of relevant markers used to discriminate myeloid populations. Expression bars below heatmaps indicate fluorescence units. **(I)** Shifts in the relative abundance of myeloid cell subsets (fold change from baseline) over the time-course of the experiment are shown. **(J)** Density plots depicting shifts in myeloid cell abundances between septic and control mice are shown (left panel) with significant differences quantified (right panel). Multiple t-tests; *=p<0.05, **=p<0.01.

High-dimensional flow cytometry was used to obtain finer resolution of the dynamics of the immune response during acute sepsis. From whole blood, this assay yielded information on twelve different immune cell subsets (CD4^+^ T cells, CD8^+^ T cells, naïve B cells, activated B cells, plasmablasts, NK cells, dendritic cells (DCs), monocytes, MDSC-like cells, mature resting neutrophils (MRNs), immature activated neutrophils (IANs), low density neutrophils (LDNs)) as well as resting versus activated platelets (**Figure 3A**)(**Supplementary Figures S3–S5**; **Supplementary Table T2**). Time-dependent shifts in immune response were noted during the sepsis time course (**Figure 3B**), with maximal disruption in immune cell abundance (as well as cytokine production and hematological parameters) reached at 12hrs post-sepsis induction (**Figure 3C**). As expected, plasma cytokine responses were the most dysregulated phenotypes observed in response to sepsis (**Figure 3C**). Neither resting nor activated platelets differed between control and septic mice at the 21hr timepoint (**Figure 3D**). Consistent with hematology data, immune dysregulation at the 21hr timepoint was primarily driven by expansion of myeloid cell subsets (**Figure 3E**, **Supplementary Figure S8**), whereas within the lymphoid subsets, we observed significant expansion of only CD4^+^ T cells in septic mice (**Figure 3F**, **Supplementary Figure S8**). Sepsis-induced emergency myelopoiesis was associated with significant reductions in the expression of several adhesion molecules (CD11b, CD44, and CD9) on myeloid cells (**Figures 3G, H**). Interestingly, decreased adhesion molecule expression was observed specifically on MDSC-like cells and LDNs (**Supplementary Figure S9**), and while all myeloid cell subsets increased in abundance in septic mice, the largest increases were observed for MDSC-like cells, LDNs, and IANs (**Figures 3I, J**).

### Sepsis is associated with rapid switch between hyperinflammatory and immunosuppressive states

**Figure 4:**
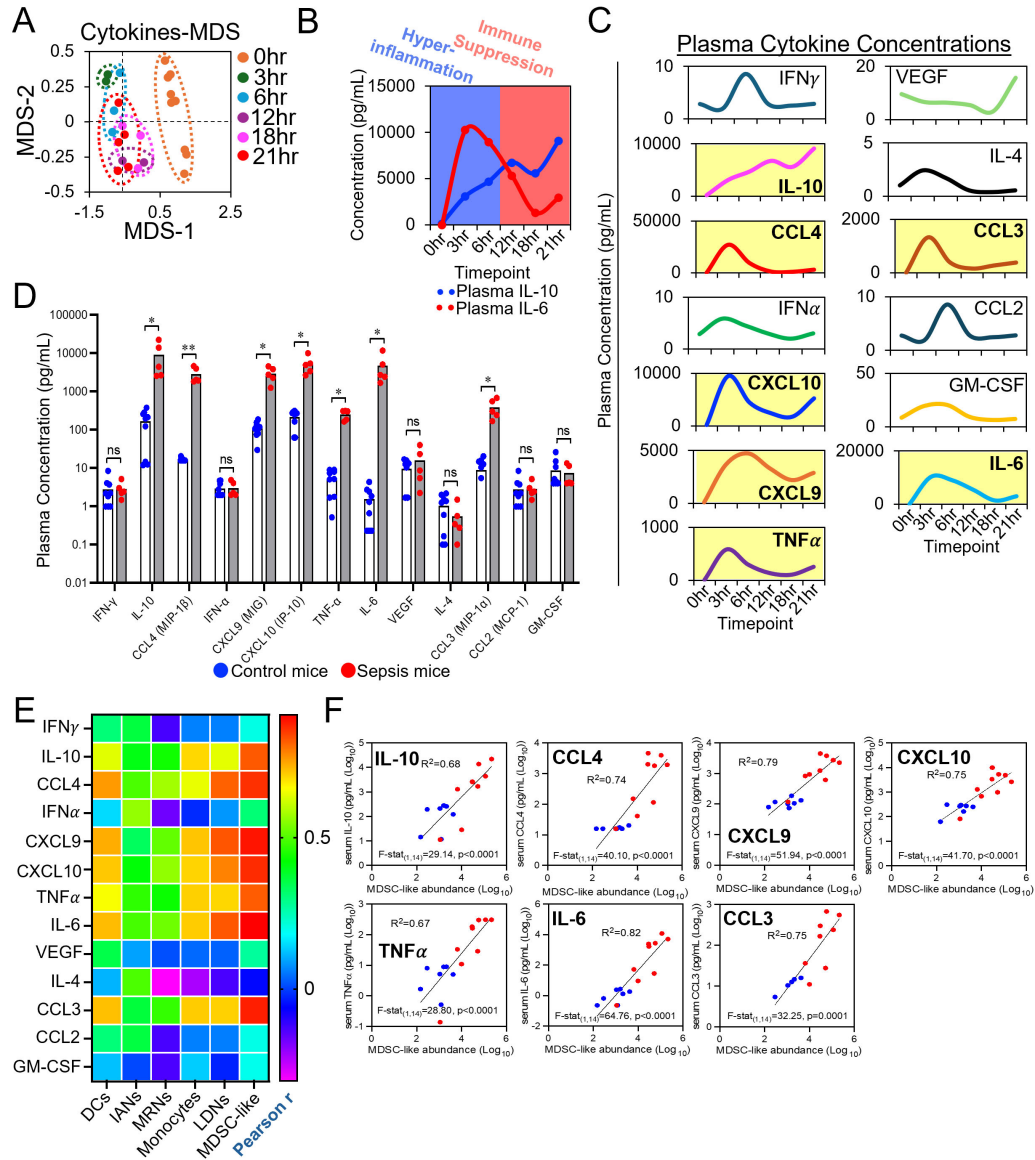
Expansion of MDSC-like cells during acute polymicrobial sepsis is associated with rapid switch from proinflammatory to immunosuppressive plasma cytokine response. **(A)** An MDS plot is shown to demonstrate the dynamics of plasma cytokine production in response to polymicrobial sepsis. **(B)** A model is provided to demonstrate the general trend observed between the production of inflammatory (e.g. IL-6) versus anti-inflammatory (e.g. IL-10) cytokines in response to an acute septic event. **(C)** Time series data is provided demonstrating flux in plasma cytokine concentrations during acute sepsis. Datasets highlighted in yellow depict cytokines whose abundance is significantly different between control and sepsis mice at experimental endpoint. **(D)** Comparisons between control and sepsis mice endpoint cytokine measurements are shown. Multiple Mann-Whitney U tests; ns=not significant, *=p<0.05, **=p<0.01. **(E)** A heatmap showing correlations between relevant plasma cytokine titers and myeloid cell subset abundance is shown (heatmap depicts Pearson r coefficients). **(F)** Correlations between relevant plasma cytokine titers and MDSC-like cell abundance are shown.

From the same cohort of mice, a flow-based multiplex assay was performed to characterized cytokine expression in septic mice over time. Time-dependent shifts in cytokine expression were observed (**Figure 4A**) with two distinct phases observed. Specifically, within the first 12hrs of sepsis induction, a hyperinflammatory cytokine response dominates the plasma cytokine pool. In stark contrast, during the final 12hrs an immunosuppressive cytokine response comes to dominate. This is easily visualized by plotting the shift between plasma IL-6 and plasma IL-10 responses over the time-course of our study (**Figure 4B**). Seven cytokines were significantly enriched over time in the plasma of septic mice (IL-10, CCL4, CXCL10, CXCL9, TNFα, CCL3, IL-6)(**Figures 4C, D**). Correlation analysis was performed between cytokine expression and myeloid cell subset abundances derived from flow cytometry analyses (**Figure 4E**). Notably, the expression of all seven cytokines were most positively correlated with the abundance of circulating MDSC-like cells (**Figures 4E, F**).

### Sepsis survival is associated with a blunted response by MDSC-like cells

**Figure 5:**
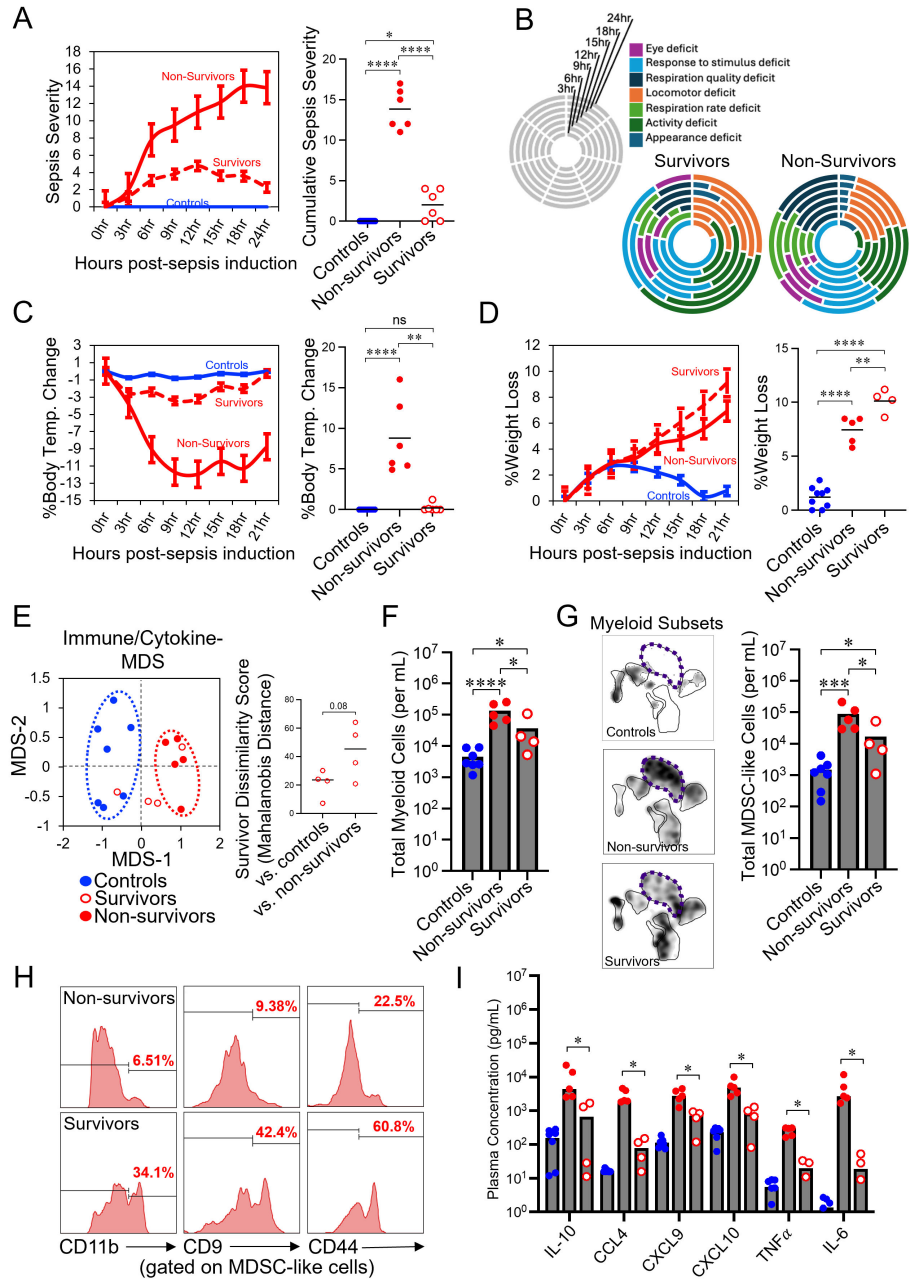
Survival from polymicrobial septic shock is associated with a blunted response by MDSC-like cells. **(A-C)** Approximately 40% of mice do not develop septic shock in response to sepsis induction as demonstrated by **(A, B)** low and improving sepsis severity scores and **(C)** body temperature changes over time. Endpoint comparisons are compared in **(A, C) (D)** Significant weight loss is observed in sepsis survivors and non-survivors. **(E)** An MDS plot is shown to demonstrate the differences in immune profiles (immune cell abundance and cytokine levels) of sepsis survivors and non-survivors at experimental endpoint. The Mahalanobis Dissimilarity Score for survivor verses control and survivor verses non-survivors is displayed. **(F)** Total myeloid cells (calculated from flow cytometry data) are compared between control mice and sepsis survivors and non-survivors. **(G)** uMAP density plots are shown to illustrate differences in myeloid cell subset abundance between survivors and non-survivors (left panel), with differences statistically compared (right panel). **(H)** Representative histograms are shown comparing the expression of adhesion molecules on myeloid cells between sepsis survivors and non-survivors. **(I)** Cytokine abundance is compared between sepsis survivors and non-survivors. **(A, C, D, F, G)** Multiple pairwise t-tests with correction; ns=not significant, *=p<0.05, **=p<0.01, ***=p<0.001, ****=p<0.0001.

In this study, nine mice underwent a septic challenge for 21hrs. Of these mice, five were deemed non-survivors, and four were deemed survivors based on severity of their symptoms (**Figures 5A–D**). Overall, survivors of sepsis showed reduced disease severity across the sepsis time course, particularly within the key indicators of mortality; respiration quality and respiration rate (**Figure 5B**). Additionally, the body temperature of sepsis survivors did not differ from control mice (**Figure 5C**). Interestingly however, both sepsis survivors and non-survivors lost significant weight in response to sepsis induction, with survivors losing more weight than non-survivors (**Figure 5D**). Immunological differences between sepsis survivors and non-survivors were also observed, with the immune response of sepsis survivors trending toward being more similar to control animals (**Figure 5E**). While both survivors and non-survivors had a significantly higher abundance of circulating myeloid cells compared to control mice (**Figure 5F**), driven mostly by expansion of MDSC-like cells (**Figure 5G**), survivors had significantly fewer cells than non-survivors (**Figures 5F, G**). Additionally, adhesion molecule expression on MDSC-like cells and LDNs was normal in survivors compared to non-survivors (**Figure 5H**, **Supplementary Figure S9**), and while the cytokine responses of survivors and non-survivors followed similar trends, non-survivors expressed significantly higher cytokine levels than survivors (**Figure 5I**, **Supplementary Figure S10A**). Additionally, correlation analysis among cytokines between survivors and non-survivors indicates that non-survivors have a more dysregulated cytokine response (i.e. weaker correlations among serum cytokine levels) compared to survivors (**Supplementary Figure 10B**). To determine if there were any differences in baseline immune parameters that might impact sepsis risk in our mice we compared circulating immune cell abundances in all animals prior to sepsis induction (i.e. T_0_ in **Figure 1A**). As expected, most immune cell subsets were equally abundant across mice. However, we did make two interesting observations. First, we found that LDN abundance was highly variable among mice and ranged between 0-35% of all myeloid cells (**Supplementary Figure S11**). More importantly, we found that mice who would go on to succumb to sepsis had significantly higher circulating LDN levels than mice that would go on to survive sepsis (**Supplementary Figure S11**).

### Blood biome analysis demonstrates that immune dysregulation and septic shock are specifically driven by outgrowth of *Enterococcus* and *Staphylococcus* in our model

**Figure 6:**
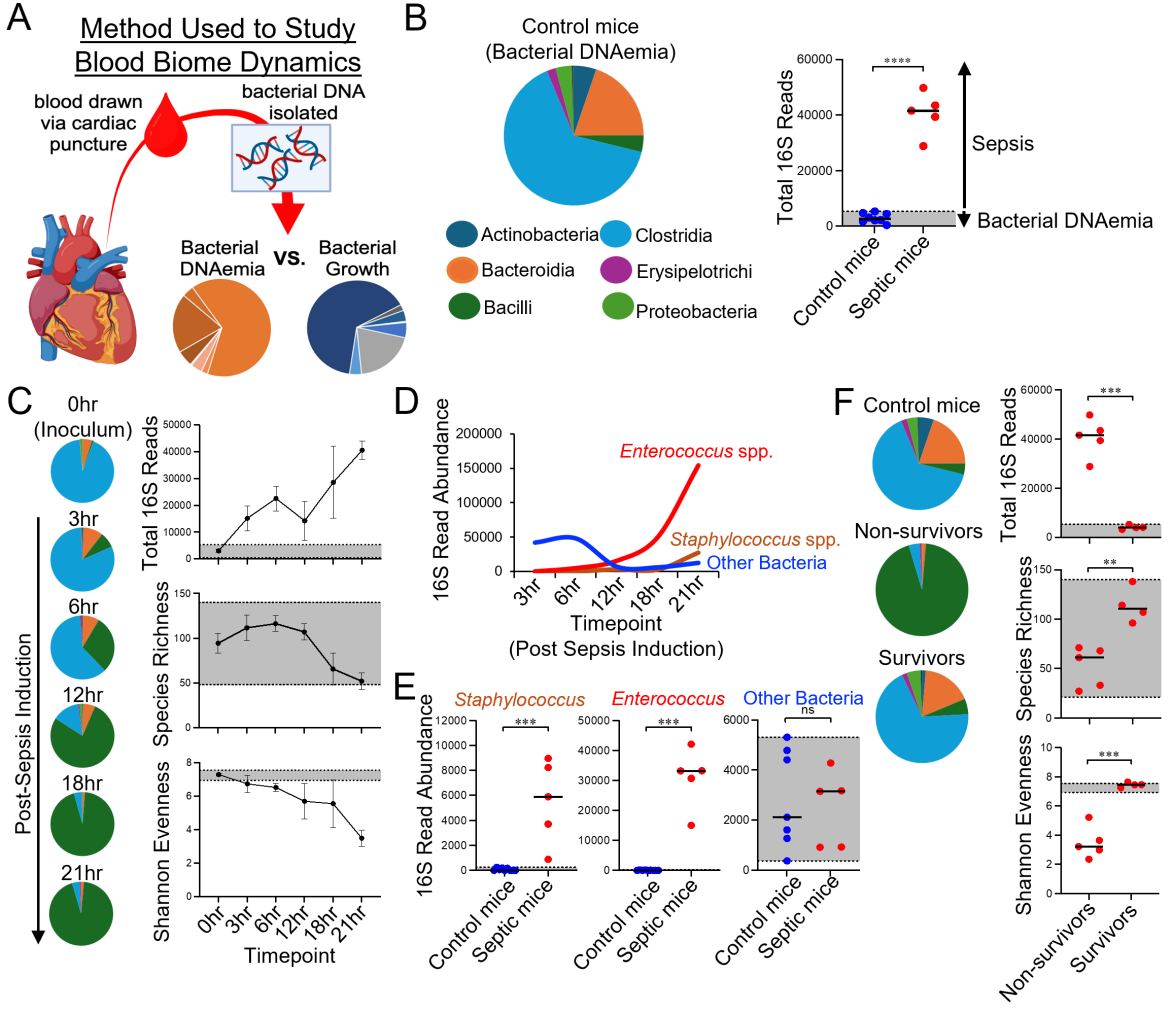
Assessment of blood biome dynamics demonstrates that polymicrobial septic shock is specifically driven by outgrowth of *Enterococcus* and *Staphylococcus*. **(A)** Schematic of our blood biome analysis that considers bacterial DNAemia versus bacterial growth using 16S rRNA profiling. **(B)** Control mice were used to delineate bacterial DNAemia. Diversity indices that fall outside the range of DNAemia (defined by shaded area) were considered useful markers of polymicrobial sepsis. **(C)** Time series data are provided to demonstrate blood biome dynamics during the course of septic shock induction. **(D)** Septic shock is specifically driven by growth of *Enterococcus* and *Staphylococcus* in the blood of septic mice. **(E)** 16S read abundance is compared between control and septic mice. **(F)** Mice that survive septic shock prohibit bacterial outgrowth in the blood. **(B, E, F)** Student’s t-test or Mann-Whitney U-test; ns=not significant, **=p<0.01, ***=p<0.001, ****=p<0.0001.

Polymicrobial sepsis is caused by the replication of two or more micro-organisms in the bloodstream. In an effort to validate that our FST approach effectively models polymicrobial sepsis, as well as to provide a novel methodology for future studies of the sepsis blood biome, we developed a novel method utilizing 16S rRNA sequencing to characterize blood microbial dynamics during the time course of acute sepsis. This approach was applied to the same animals utilized in the phenotyping experiments described above. With the exception of portal vein flow that drains blood from the gut (and is consequently enriched in microbial products and microbes), blood is considered sterile. However, bacterial DNA is readily detectable in mouse and human blood samples (bacterial ‘DNAemia’) and represents a confounding source of DNA that must be accounted for in sequence-based approaches to study the blood biome (**Figure 6A**). Indeed, bacterial DNAemia in control mice largely reflects the typical phylogenetic breakdown of the murine fecal microbiome with enrichment of sequences representing the Firmicutes (mostly Clostridia) and Bacteroidia phyla (**Figure 6B**). To control for confounding bacterial DNAemia in mice, we performed a subtraction method whereby OTU read abundances in septic mice were adjusted (for each OTU we calculated: OTU_sepsis_ abundance-OTU_DNAemia_ abundance=adjusted OTU_sepsis_ abundance). As demonstrated by the highly significant increase in total 16S reads obtained from the blood of septic versus control mice, bacterial DNAemia has a negligible contribution to the total 16S reads obtained in septic mice (**Figure 6B**). Time series data of septic mice demonstrated a clear stepwise outgrowth of bacteria (primarily Bacilli) within the blood of septic mice as demonstrated by the concomitant increase in total 16S reads (**Figure 6C**) and decrease in species richness and sequence evenness (**Figure 6C**). At 3hrs post-sepsis induction, high abundance of a variety of bacterial species were observed in the blood of septic mice (**Figure 6D**). However, time series analysis revealed that over time two specific species of bacteria outgrew in the blood of septic mice; an unidentified *Enterococcus* species and an unidentified *Staphylococcus* species (**Figure 6D**). Neither of these species were detected in the bacterial DNAemia of control mice (**Figure 6E**). This data confirms that FST results in polymicrobial sepsis; the dynamics of which can be studied using our method. Finally, bacterial outgrowth was controlled in mice that recovered from sepsis induction (I.e. total 16S abundance, species richness, and sequence pool evenness within the ranges of bacterial DNAemia)(**Figure 6F**).

## Discussion

Polymicrobial infections are particularly life-threatening compared to monomicrobial infections. Though there have been conflicting reports on the associated mortality of polymicrobial verses monomicrobial sepsis, many studies demonstrate increased risks to patients with polymicrobial infections. For example, an observational study conducted between 2013 and 2019 at a single hospital in China found that patients with polymicrobial infections had longer hospital stays, a two-fold higher risk of developing septic shock, and increased mortality rates (40). Vasopressors are a common line of treatment during septic shock, however, these vasopressors lower the patient’s ability to adequately respond to sepsis (41). Additionally, polymicrobial sepsis has been shown to lead to refractory shock, which in turn necessitates the use of more (or higher doses of) vasopressors (42). In a study conducted on data collected by the Korean Sepsis Alliance Registry between 2019 and 2021, it was found that polymicrobial sepsis was associated with increased crude mortality, increased Sequential Organ Failure Assessment (SOFA) scores, and increased serum lactate levels (43). However, this study concludes that this discrepancy in hospital mortality is not due to the polymicrobial sepsis itself, but rather to inappropriate or delayed use of antibiotics (43). In fact, it has been shown that in patients with septic shock, every hour that antibiotic treatment is delayed within the first three hours of admission the risk of mortality increases by 35% (44). There is also evidence to indicate that polymicrobial sepsis is frequently missed due to the fact that the microbial ecology of sepsis is poorly understood (26). This lack of understanding of the microbial dynamics of sepsis and the differential way it informs treatment emphasizes the need for more in depth studies of the microbial ecology of acute polymicrobial septic shock. These points were a major motivating factor for the current study.

The FST model of sepsis replicates established sepsis phenotypes in humans while avoiding confounding variables introduced by other methods by standardizing the dose and composition of the microbial inoculum. The most common established animal model of polymicrobial sepsis is the cecal loop puncture (CLP) model. As we have observed, The CLP model also results in the outgrowth of enteric bacteria in the blood beginning around six hours post sepsis induction as well as sepsis symptoms around twelve hours post septic event (45). However, this model is limited for several reasons including differences in cecal anatomy between mouse strains, its induction of intestinal necrosis, and the variance in experimental results caused by the inability to standardize dose and composition of the microbial inoculum (30). By contrast, the FST model delivers a defined polymicrobial inoculum into the peritoneal cavity without causing any physical tissue damage, allowing for greater reproducibility.

Sepsis causes rapid immune dysregulation defined by an early hyper-inflammatory response followed by immune suppression (46). During the hyperinflammatory phase, the presence of microbes and dead/dying cells elicits the rapid release of proinflammatory cytokines that instigates emergency hematopoiesis (47). During periods of stress and high demand, such as during emergency hematopoiesis, HSCs transition from glycolysis to oxidative phosphorylation (48, 49). This surge in oxidative phosphorylation may contribute to increased fumarate production, even transiently. Mitochondrial dysfunction during oxidative phosphorylation has been linked to increased mortality in septic patients (50). Mitochondrial stress also results in the generation of 2SC, an aberrant byproduct of the citric acid cycle (51, 52). We observed elevated levels of 2SC in the blood of non-survivors compared to survivors and uninfected controls. Mortality may therefore be linked to whether or not an individual enters into a state of emergency hematopoiesis. Emerging evidence indicates that sepsis-induced emergency hematopoiesis markedly influences myeloid cell heterogeneity (and consequently function) in circulation that can persist long after the resolution of the initial septic event (18). Immune paralysis is a term used to describe the persistence of an immunosuppressive state in sepsis survivors, and this phenotype is likely (in part) reflective of this effect. In our study and consistent with emergency hematopoiesis, we observe an initial wave of immune depletion (0-12hrs post induction) followed by a second wave of myeloid cell expansion (12-21hrs post induction). This biphasic response coincides with a shift in the observed cytokine storm from one dominated by proinflammatory cytokines (IL-6, TNFα) and chemokines (CCL2, CL3, CCL4, CXCL9, CXCL10) to one dominated by the immunosuppressive cytokine (IL-10). The emergence of immunosuppressive IL-10 responses corresponded to the emergence of MDSC-like cells during the time-course of infection. An important limitation we acknowledge is that we have not directly measured functional variation in myeloid cell subsets. This is particularly important for accurately identifying MDSCs, which is why we refer to our cells as “MDSC-like” throughout our results section. Future studies incorporating functional markers into our flow panel (e.g. ARG1 and/or iNOS) and downstream single cell RNA sequencing of sort-purified subsets will be necessary for validating these cells as true MDSCs.

The term ‘blood biome’ refers to the collection of microbes found living in circulation. While blood is typically sterile, bacterial DNA is routinely found in the circulation of humans and mice. The presence of bacterial DNA in blood has been termed bacterial DNAemia and is a confounder to the study of the ‘blood biome’. We adopted this terminology, quantified bacterial DNAemia in mice, and used this to demonstrate that the blood biome data we generated reflects true bacterial replication in blood. Importantly, our 16S analysis validated the polymicrobial nature of sepsis in our model by revealing outgrowth of two bacterial species in the blood of septic mice; an unidentified *Enterococcus* species and an unidentified *Staphylococcus* species. Furthermore, the outgrowth of *Enterococcus* and *Staphylococcus* species observed in our model shows promising translational relevance to humans. *Enterococcus* and *Staphylococcus* species have commonly been implicated as causative agents in septic shock, especially when sepsis is derived from the intestines (53–55). Previous studies have shown that MDSCs are upregulated in the context of sepsis and that MDSCs are more significantly impacted when the source of a septic infection is a gram-positive bacterium (56). Thus, the FST model may be particularly effective at modeling acute MDSC responses (after further functional validation of these cells). A caveat to our study is that a single microbial inoculum was used to induce sepsis. Thus, the impact of compositional variation in the microbiome among individuals on sepsis risk/severity was not assessed, but can be easily modeled in future experiments by inoculating individuals with their own microbiomes. Additionally, we considered bacterial DNA in non-septic mice as sterile, and 16S sequence read abundance above this level as indicative of active microbial replication. Future studies should validate that the blood of non-septic mice are microbiologically clean and that actively growing bacteria can be cultured from the blood of septic mice.

Post-sepsis induction, we find that sepsis dysregulates the expression of several adhesion molecules on MDSC-like cells and LDNs in mice. Loss of CD9 is a notable phenotype for several reasons. CD9 is a member of the tetraspanin family of proteins that are known to influence a variety of cellular responses by immune cells (57). CD9, along with other tetraspanins (e.g. CD63 and CD81), are also markers of exosomes. Previous work implies that loss of CD9 surface expression may be indicative of myeloid cell activation status and/or differential polarization. From the perspective of activation, INFγ reduces CD9 expression by macrophages (58), and lower CD9 expression is associated with protease secretion (59). In another study, CD9 depletion in macrophages was also shown to sensitize them to LPS stimulation resulting in greater TNFα synthesis (60). From the perspective of polarization, loss of CD9 on circulating monocytes could reflect a shift in monocyte development in the bone marrow and/or polarization in response to stimuli in peripheral tissues. In humans, CD9 expression is inversely related to CD16 expression in monocytes (61). CD14^dim^CD16^high^ monocytes are termed non-classical monocytes that represent a minor fraction of total circulating monocytes in healthy humans. However, emergency hematopoiesis induced by sepsis results in an acute and dynamic redistribution of circulating monocyte subsets with enrichment of non-classical monocytes in sepsis patients (62). Thus, it is reasonable to assume that CD9 expression would be reduced on average in circulating myeloid cells in these patients (though to our knowledge this has not been studied). Previous work has also shown that monocytes undergo functional reprogramming during sepsis with a strong bias toward endocytosis (63), and endocytosis has been shown to abolish CD9 surface expression on cells (64). Thus, sepsis can destabilize the expression of surface proteins by regulating myeloid cell polarization, which may explain the common observation of reduced surface expression of other proteins on myeloid cells in sepsis patients (e.g. HLA-DR). Ultimately, we conclude that the observed CD9 phenotype likely represents a developmental shift induced by emergency hematopoiesis that favors enrichment of a functionally unique monocyte population in circulation. Future studies performing paired analysis of myelopoiesis in the bone marrow will be crucial for understanding the factors driving this developmental switch.

Non-classical monocytes, MDSCs, and LDNs can exert immunosuppressive functions and therefore all are capable of contributing to the immune paralysis observed in sepsis patients. Sepsis patients have been shown to be enriched for all three cell types (20, 62, 65). While a recent study using single cell RNA sequencing has demonstrated that sepsis can induce “functional reprogramming” of monocytes (63), biasing them toward a repair phenotype, they did not consider cellular heterogeneity and instead performed bulk monocyte analysis. While important observations were gleaned from this work, it was limited as the compartmentalization of functions among specific cell subsets was not able to be studied. A more recent study using CITE-seq characterized functional diversity within MDSCs and identified a unique developmental pathway and MDSC subset enriched in sepsis patients with poor clinical outcomes (18). Thus, it is likely that sepsis-induced emergency hematopoiesis results in long-lasting perturbations to myelopoiesis that explain the chronic immunosuppression observed in human sepsis survivors.

Interestingly, our experimental method identifies prognostic biomarkers that predict sepsis outcome before sepsis induction. Specifically, we observed that mice that would go on to survive an acute septic event had fewer LDNs in their circulation prior to sepsis induction. This result supports the following interpretations. First, circulating levels of these cell types may be prognostic of sepsis risk in humans, and indeed, higher LDN abundance has been associated with significantly elevated risk of secondary bacterial infection and mortality in human sepsis patients (65). Second, having fewer LDNs at the initiation of a septic event may delay the onset of immunosuppression to allow for microbial clearance of offending microbes. Collectively, results from our experiments suggest that suppression of emergency myelopoiesis may protect against septic shock.

Our experimental method also identifies biomarkers that predict sepsis outcome during a septic event. As expected, survival from sepsis was associated with less bacterial growth in the blood and a weaker ensuing immune response. What is interesting is the observation we make that, post sepsis induction, weight loss becomes a predictive biomarker of sepsis survival. In our experiment, we observed that sepsis survivors lost more weight than non-survivors, which cannot be accounted for by duration of the septic event as both groups of mice were euthanized at the same time-point post-sepsis induction (21hrs). Increased weight loss among human sepsis survivors has also been documented, and it has been speculated that this is due to fluid retention in non-survivors (66). Additionally, the same phenotype we describe has also been shown in a previous study using the CLP mouse model of sepsis; non-survivors lost significantly less weight during the acute phase of infection compared to survivors (67). In addition to fluid retention, we would like to put forth the hypothesis that metabolism is a possible prognostic indicator of sepsis risk and severity. Interestingly, and in support of this hypothesis, it has been widely reported that in sepsis there is an ‘obesity paradox’, where overweight and obese patients have higher sepsis survival rates compared to average weight individuals (68–73). This is due to a variety of factors, but at face value it supports our contention that metabolism, and factors regulating it like diet, may have substantial impacts on sepsis risk/severity that should be explored in more depth. Indeed, sepsis results in a hypermetabolic response with supra-physiological energy requirements (74). The obesity-paradox in sepsis may be explained simply by the increased ability of obese individuals to meet these energetic demands. More work on how metabolic tone influences sepsis risk is warranted. Of note, our experimental model is acute, requiring mice to be euthanized within 24 hours of sepsis induction. This limits our ability to study the prognostic role of these potential biomarkers in the context of chronic sepsis. To address this, future work can include less severe models of sepsis (e.g. the CLP model).

The merits of the work we describe in this study lie in the approaches we have developed to comprehensively study the pathogenesis of acute polymicrobial sepsis. In this study, we performed simultaneous measurements of hematological, immunological, microbiological, and physiological readouts to assess the cellular and microbial dynamics of an acute septic event. Importantly, we have demonstrated that our approach effectively captures both elements of the immune dysregulation caused by sepsis; characterized by the dynamic switch between hyperinflammatory and immunosuppressive immune responses. Notably, we were also able to use this approach to quantify shifts in the immune status of mice that preceded severe symptoms caused by sepsis, as well as among mice prior to sepsis induction, highlighting the potential of our approach to identify predictive biomarkers of sepsis severity and risk. We also detail a new approach that can be employed to study blood biome dynamics during an acute septic event, which has never been described before and is a major gap in knowledge. This approach provides a useful (and adaptable) platform for future studies seeking to understand (and ultimately protect) humans from acute septic shock.

## Data Availability

Raw experimental data sets were made available through Dryad Data Repository (DOI: 10.5061/dryad.r7sqv9srz). All 16S rRNA were made available through the NCBI SRA repository (PRJNA1356527).
