# Correction: Multiplexed longitudinal analysis of the cellular and microbial dynamics of acute polymicrobial sepsis in mice

**DOI:** 10.3389/fimmu.2026.1794085

**Published:** 2026-02-12

**Authors:** Tori E. Peacock, Kenny Johnson, Abhinav R. Cheedipudi, Ahmed D. Mohammed, Ryan A. W. Ball, J. Hunter Cox, Savannah K. Pender, Amy Jolly, Kandy T. Velázquez, Jay Potts, Angela Murphy, Norma Frizzell, Colin Evans, Jason L. Kubinak

**Affiliations:** 1 Department of Pathology, Microbiology, and Immunology, School of Medicine, University of South Carolina, Columbia, SC, United States; 2 Department of Pharmacology, Physiology, and Neuroscience, School of Medicine, University of South Carolina, Columbia, SC, United States; 3 Department of Cell Biology and Anatomy, School of Medicine, University of South Carolina, Columbia, SC, United States

**Keywords:** sepsis, blood biome, septic shock, spectral flow cytometry, emergency myelopoiesis

File Data Sheet #1 was erroneously published with the original version of this paper. The file has now been replaced.

The original version of this article has been updated.

