# Supplementary material for: Multiplexed longitudinal analysis of the cellular and microbial dynamics of acute polymicrobial sepsis in mice
Source: Front Immunol. 2025 Dec 3;16:1682451. doi: 10.3389/fimmu.2025.1682451 (PMC12710661; doi:10.3389/fimmu.2025.1682451)
Supplement: Supplementary file 1 [file DataSheet1.pdf]

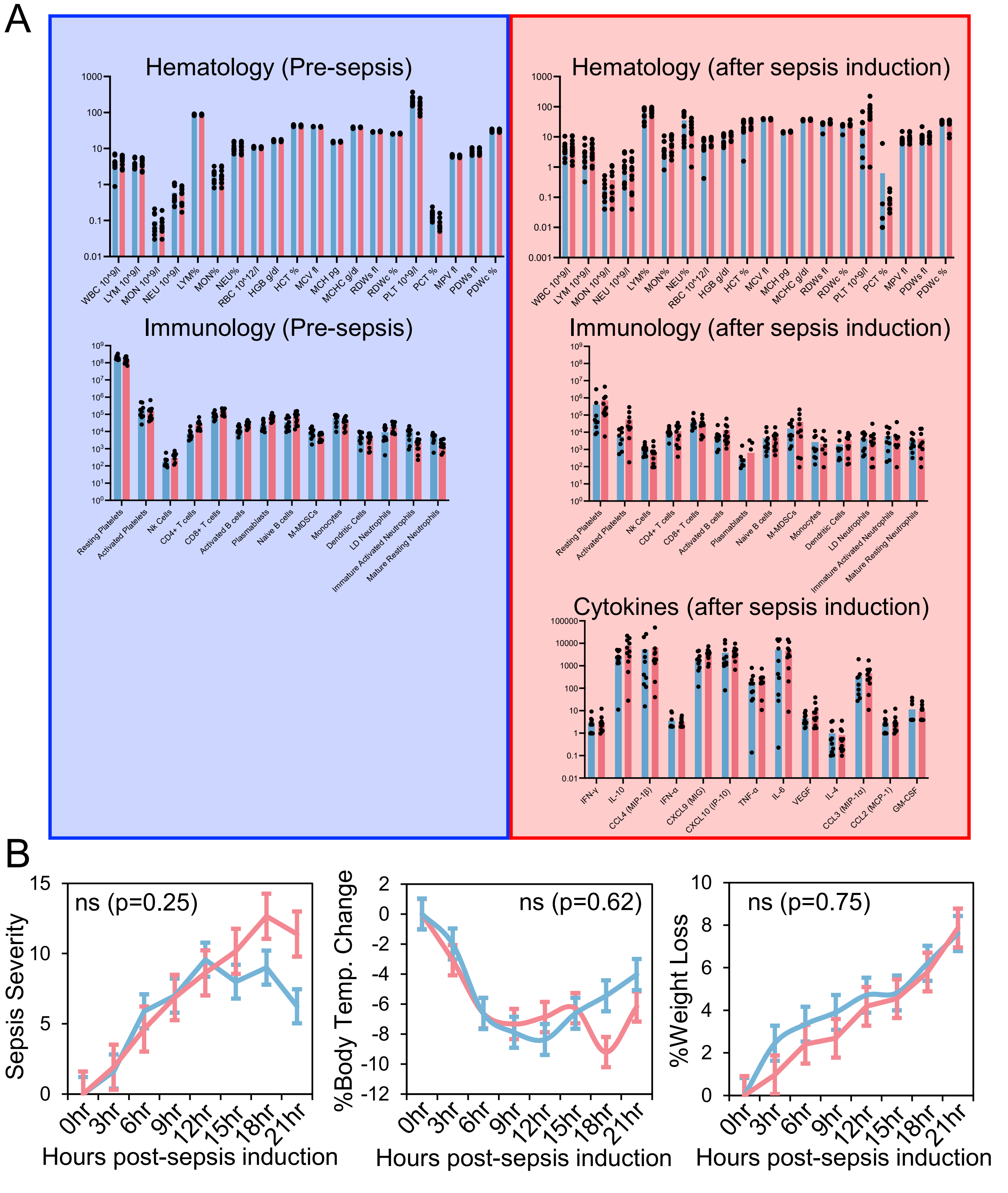

Supplementary Fig S1. Sex does not significantly impact hematology values, immune cell abundance, cytokine responses, or disease severity in our model. (A) After adjusting for multiple comparisons, no significant differences between males and females were observed in any hematological, immune, or cytokine readout. **(B)** No significant differences were observed between male and female mice in our three estimates of disease severity. Student's t-test was performed on endpoint data (ns=not significant, p-value shown). (A and B) Data shown represents the same male and female mice prior to (blue panel), and after (red panel), sepsis induction. Cytokine responses were not measured at initial timepoints.

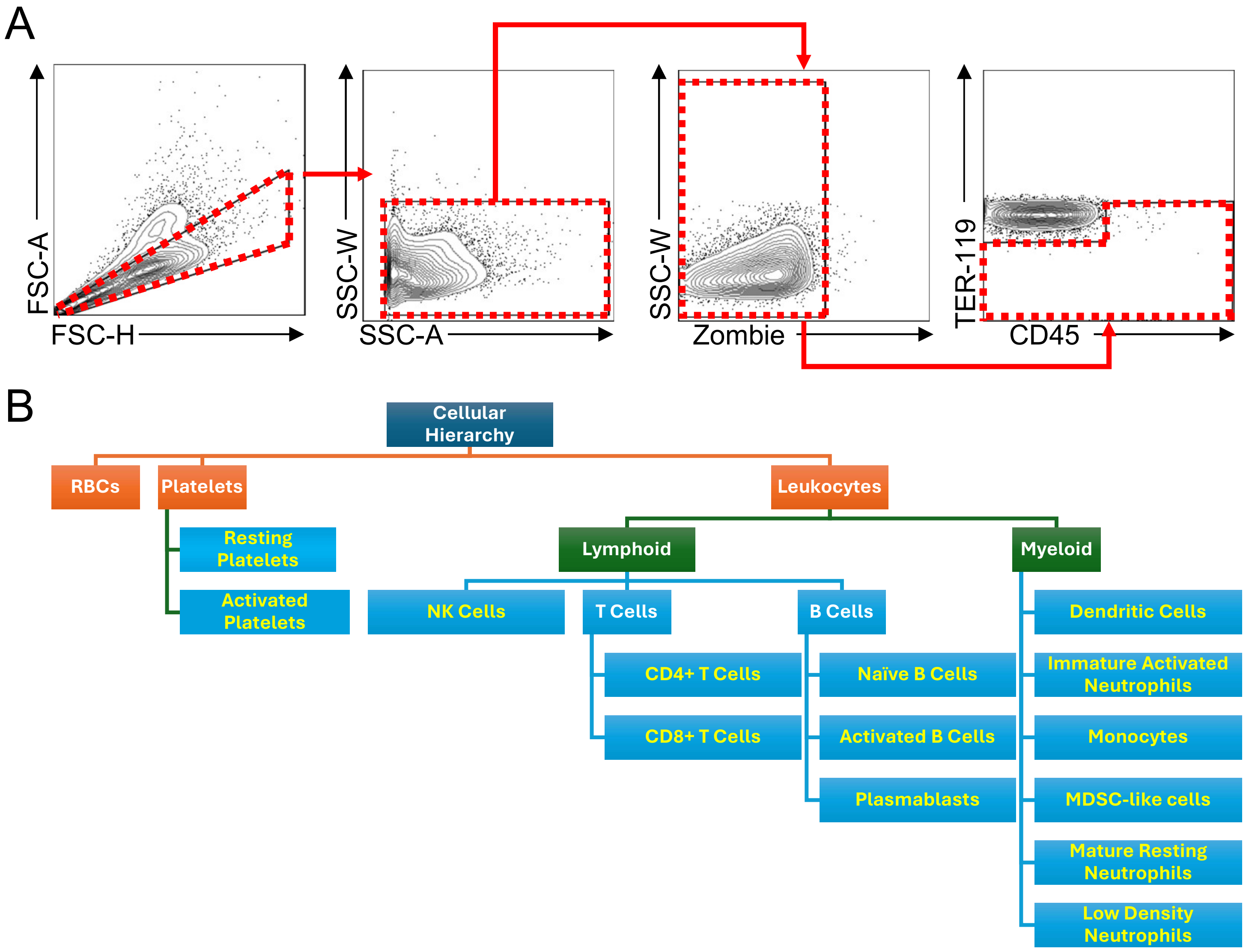

Supplementary Fig S2. FACSDiscover S8 instrument setup and panel design rationale. (A) On-instrument enrichment strategy to minimize RBC event contribution to downstream analyses is shown. Live (Zombie^{-}) ter119^{-}CD45$^{+/-}$ singlet events were collected on the FACSDiscover S8 for inclusion in downstream analysis. **(B)** Overview of blood cell heterogeneity that can be appreciated with our approach. 14 terminal populations enumerated with this assay are highlighted with yellow font.

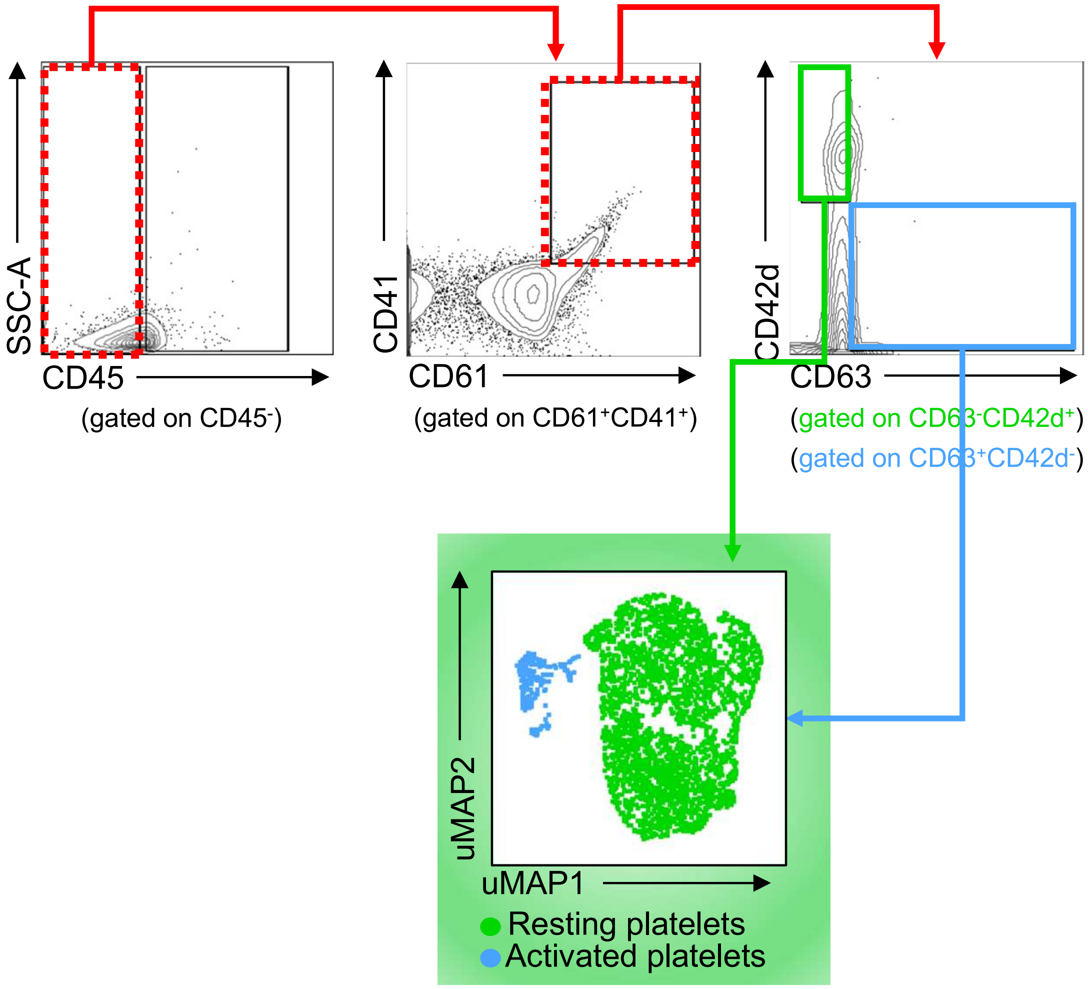

Supplementary Fig S3. Gating rubric used to identify platelets subsets. From total collected events from the FACSDiscover S8 instrument we gated on CD45$^-$CD41$^+$CD61$^+$ events to identify platelets. Within the platelet population (CD45$^-$CD41$^+$CD61$^+$), we gated on CD63$^-$CD42d$^+$ to identify **resting platelets** and CD63$^+$CD42d$^-$ to identify **activated platelets**. Events from terminal populations were concatenated for uMAP projection and the creation of treatment density plots shown in main figures. Drilling down for subset enumeration is indicated by **red hashed lines**.

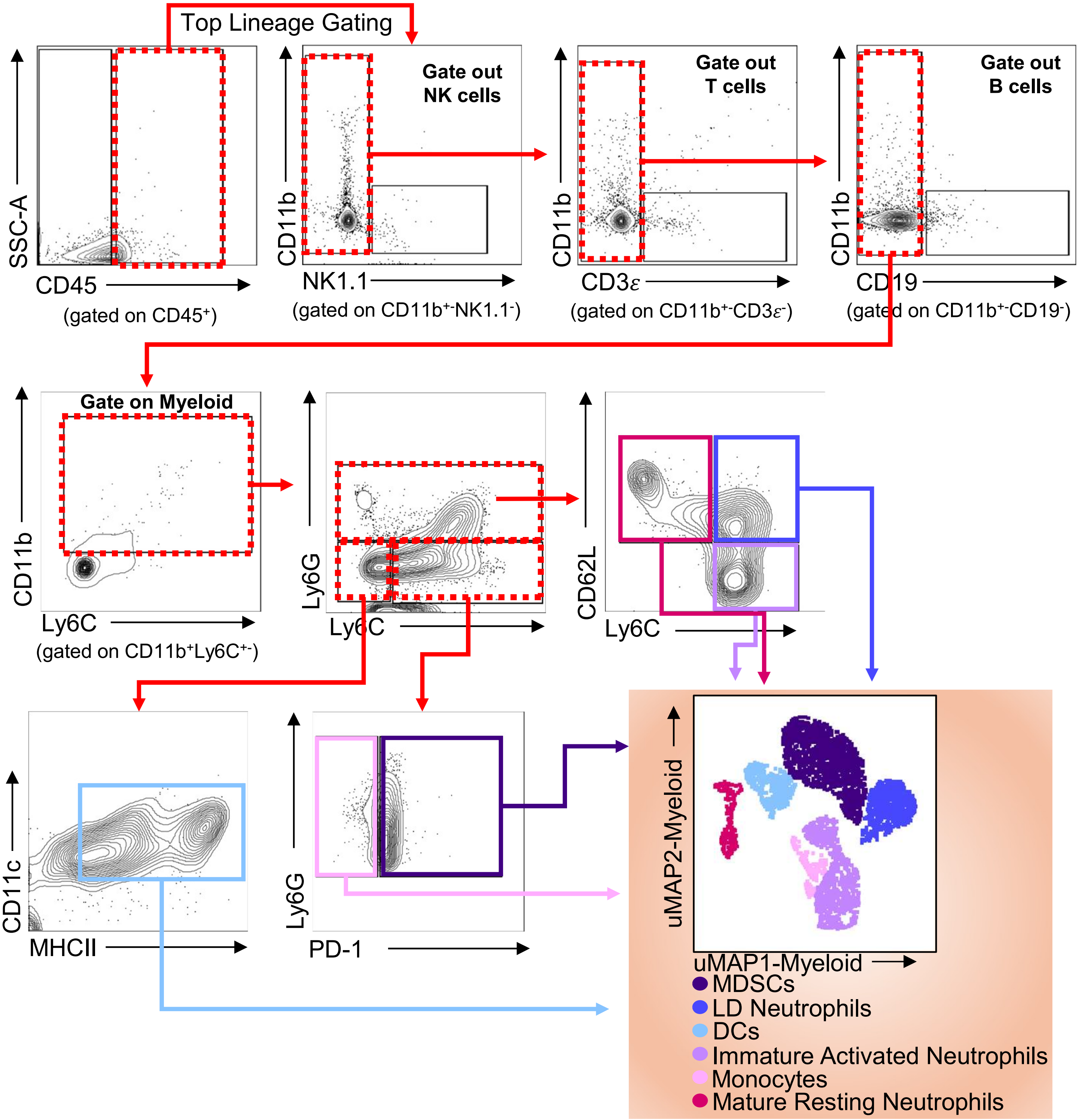

Supplementary Fig S4. Gating rubric used to identify myeloid subsets. From total collected events from the FACSDiscover S8 instrument we gated on CD45$^+$CD41$^-$ events to identify immune cells. Within the immune cell population (CD45$^+$CD41$^-$), we performed top lineage gating via successive negative gates off of the CD11b^{+-} population to gate out NK cells (NK1.1$^+$), T cells (CD3ε^-) , and B cells (CD19$^+$). From the CD11b^{+-}NK1.1$^-$CD3ε^-CD19$^-$ population, we gated on CD11b$^+$Ly6C^{+-} to drill down on myeloid cells. Within the myeloid cell population, we gated on the Ly6C$^-$Ly6G$^-$ population to identify **dendritic cells** (**DCs**)(CD11c$^+$MHCII$^+$) and within the Ly6C$^+$Ly6G$^-$ population to identify **myeloid derived suppressor like cells** (**MDSCs**)(PD-1+) and **monocytes** (PD-1-). We gated on the Ly6C^{-+}Ly6G^{+-} population to identify **Low Density Neutrophils** (**LD Neutrophils**) (Ly6C$^+$CD62L$^+$) and **Immature Activated Neutrophils** (Ly6C$^+$CD62L$^-$), and **Mature Resting Neutrophils** (Ly6C$^-$CD62L$^+$). Events from terminal populations were concatenated for uMAP projection and the creation of treatment density plots shown in main figures. Drilling down for subset enumeration is indicated by **red hashed lines**.

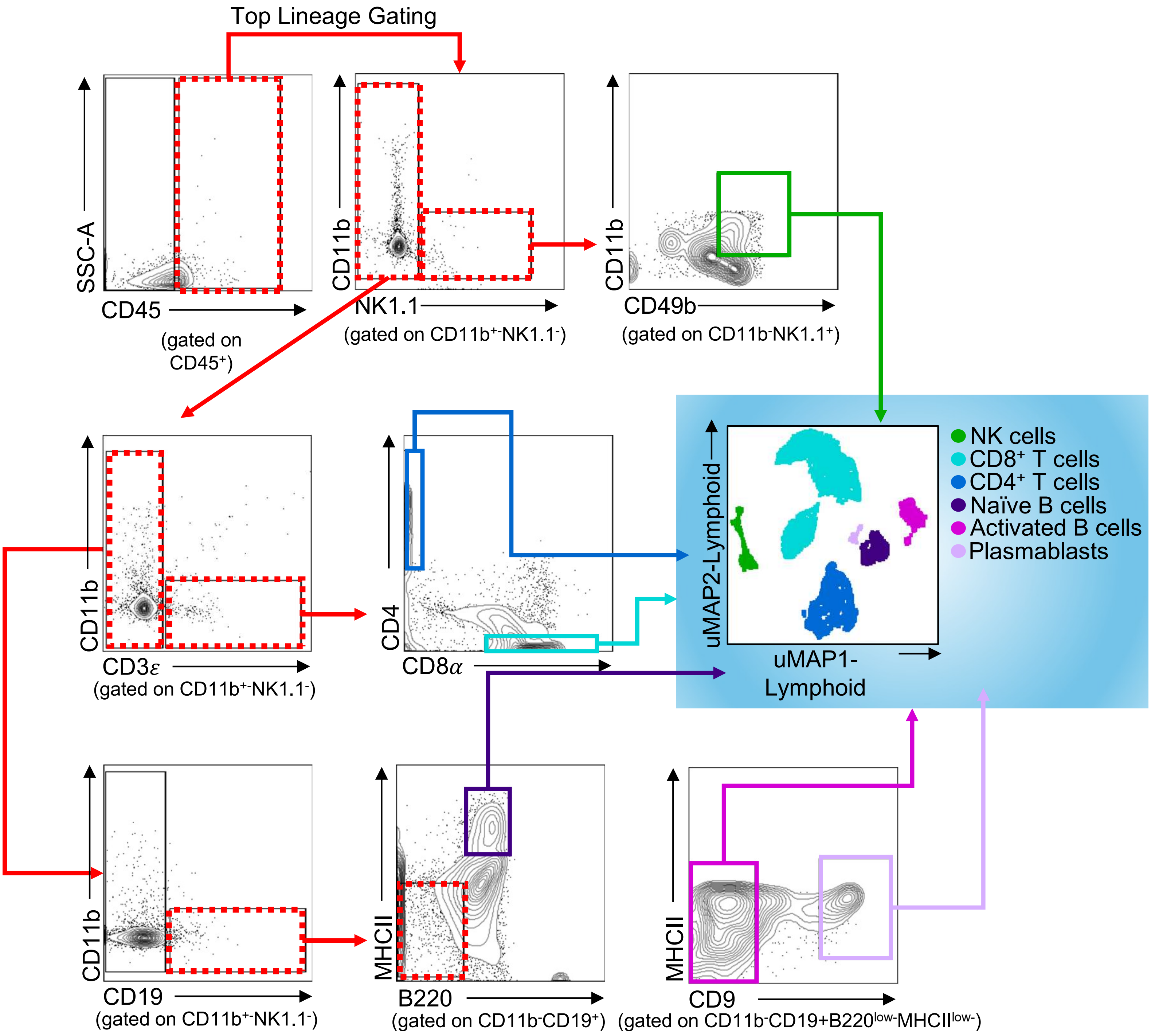

Supplementary Fig S5. Gating rubric used to identify lymphoid subsets. From total collected events from the FACSDiscover S8 instrument we gated on $CD45^{+}CD41^{-}$ events to identify immune cells. Within the immune cell population ($CD45^{+}CD41^{-}$), we performed top lineage gating off of the $CD11b^{+-}$ population to gate out NK1.1 cells ($NK1.1^{+}$). Within the $NK1.1^{+}$ population we then gated on $CD11b^{low}CD49b^{+}$ to identify **NK cells**. From the $CD11b^{+-}NK1.1^{-}$ population we then gated on $CD3\varepsilon^{+}$ events. Within the $CD3\varepsilon^{+}$ population we gated on $CD4^{+}CD8^{-}$ to identify **$CD4^{+}$ T helper cells** and $CD4^{-}CD8^{+}$ to identify **$CD8^{+}$ cytotoxic T cells**. From the $CD11b^{+-}NK1.1^{-}CD3\varepsilon^{-}$ population, we gated on $B220^{+}MHCII^{+}$ events to identify **naïve B cells**. From the $B220^{low-}MHCII^{low-}$ population, we gated on the $CD9^{+}$ population to identify **plasmablasts** and the $CD9^{-}$ population to identify **activated B cells**. Events from terminal populations were concatenated for uMAP projection and the creation of treatment density plots shown in main figures. Drilling down for subset enumeration is indicated by **red hashed lines**.

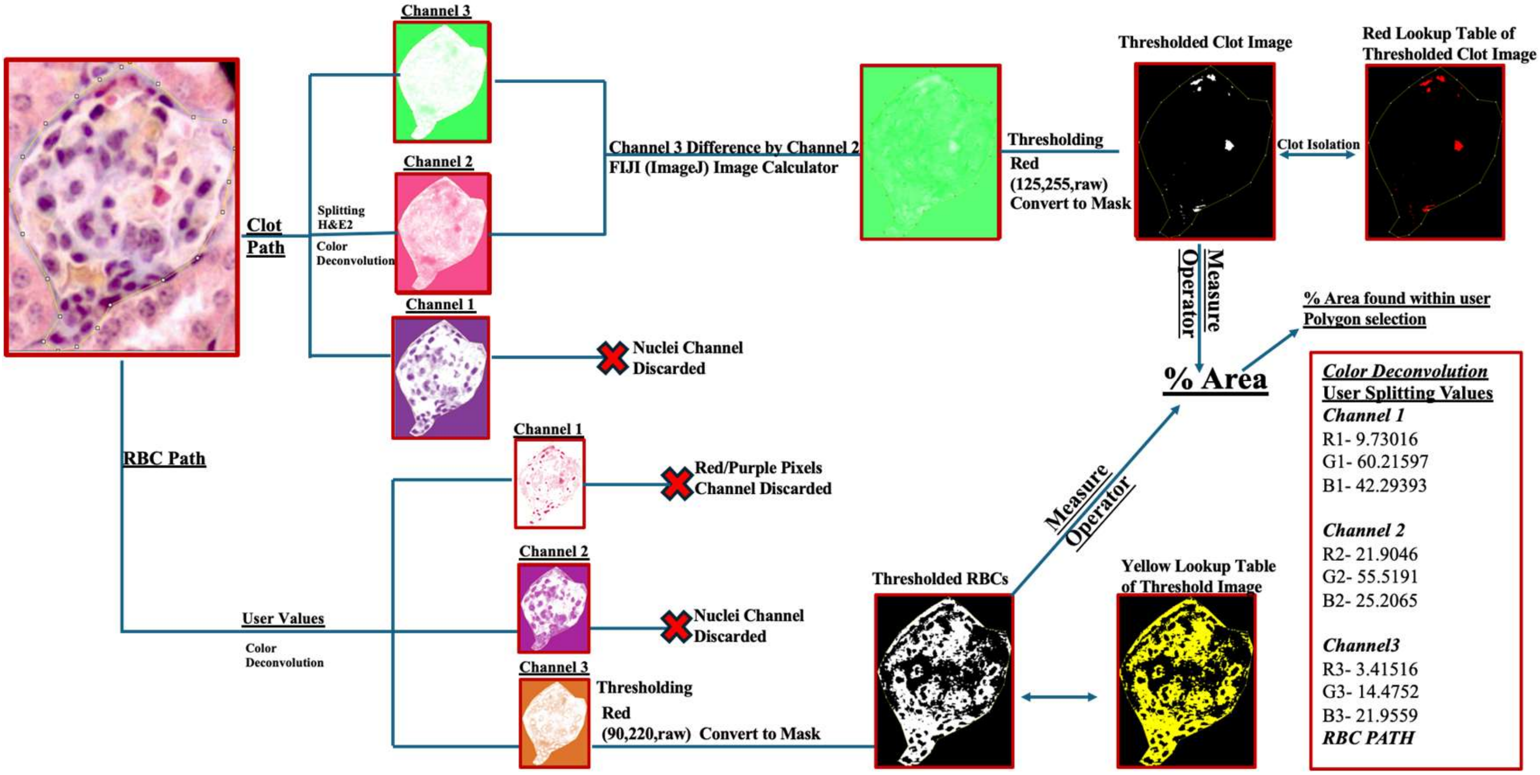

Supplementary Fig S6. Representative decision tree regarding automated assessment of MSB-stained kidney glomeruli. Image J Macro programming language was used to automate the glomeruli selection and fibrin clot area from the 10x Revolve Kidney Cortex images. The glomeruli were selected using the polygon selection tool in Image J. The Bowman's Capsule was used as the landmark for the glomeruli selection. The specific region selection coordinates were saved using the ROI manager tool (the raw .zip files were saved for each mouse). Each mouse's glomeruli were saved in the respective subfolders. Each image was opened, the respective region from the ROI manager was placed on the image, everything outside of the polygon region was cleared, Image J's color deconvolution's H&E2 splitting technique was placed on the image, the Image Calculator (in the process tab) was loaded, a new image was generated using the operation channel three difference channel two, the new processed image was thresholded and converted to mask using red thresholding in the regions (125, 255, raw), and the measure operator was used to gather the area of the fibrin clot (percent area) and the area of the glomeruli within the respective ROI region.

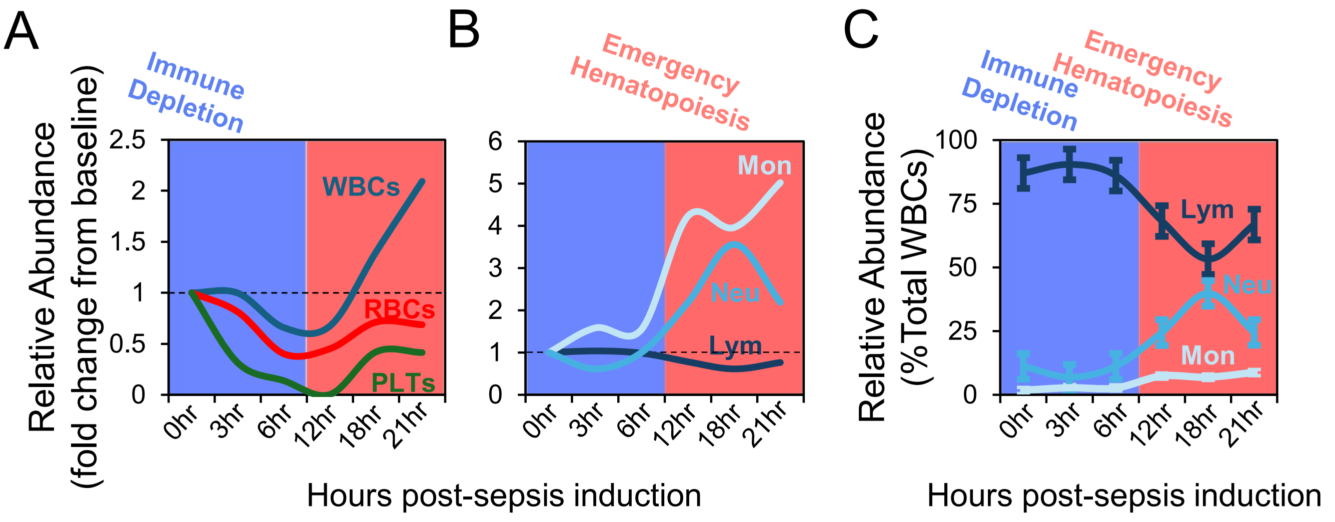

Supplementary Figure S7. Immune depletion in septic mice is driven by lymphopenia and emergency hematopoiesis is driven by expansion of myeloid cells. (A and B) Shifts in the relative abundance of major blood cell (A) and immune cell (B) subsets (fold change from baseline) over the time-course of the experiment are shown. **(C)** The relative abundance of WBC subsets are shown as a percentage of total WBCs.

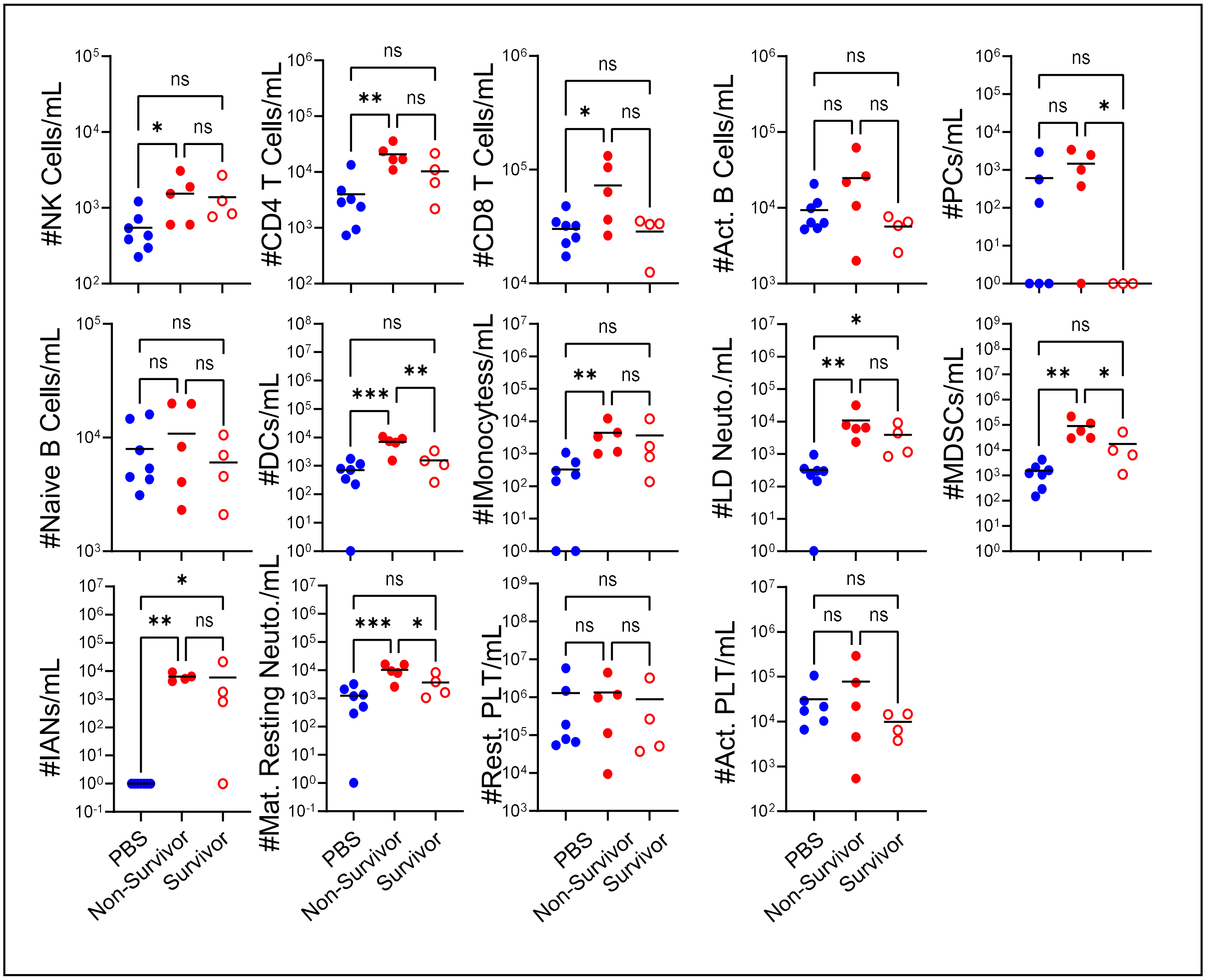

Supplementary Fig S8. Pairwise comparisons of flow cytometry data between control and septic mice at 21hrs post sepsis induction. Uncorrected Fisher's LSD or Kruskal-Wallis test; ns=not significant (p>0.05), *=p<0.05, **=p<0.01, ***=p<0.001.

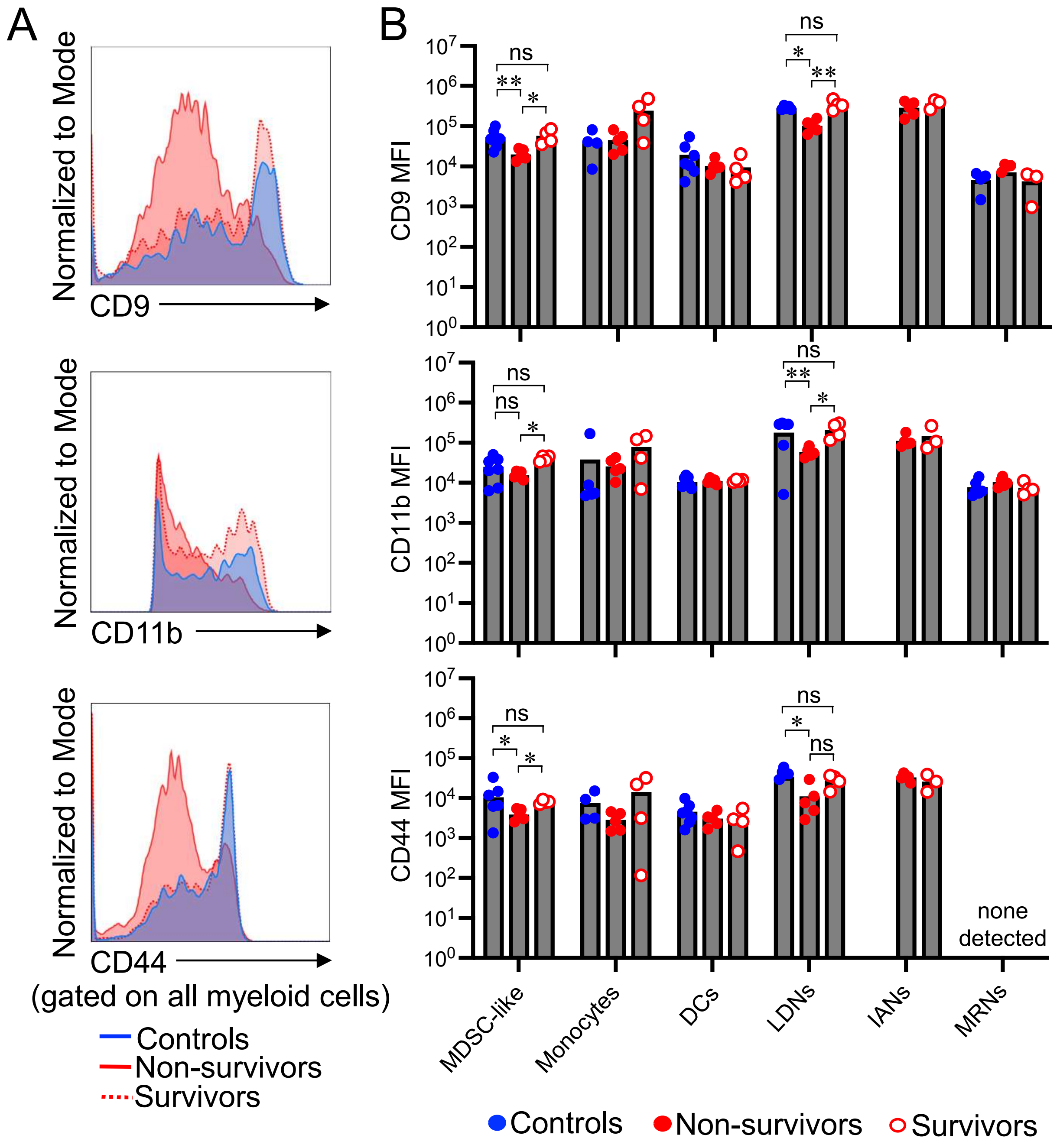

Supplementary Fig S9. Adhesion molecule expression (MFI) on myeloid subsets. (A) Representative histograms of total myeloid cells are shown for each adhesion molecule marker and highlight the reductions in adhesion molecule expression specifically on myeloid cells from non-survivors. **(B)** Pairwise comparisons showing significant difference in adhesion molecule expression (mean fluorescent intensity) on myeloid cell subsets. Kruskal-Wallis test; ns=not significant, *=p<0.05, **=p<0.01.

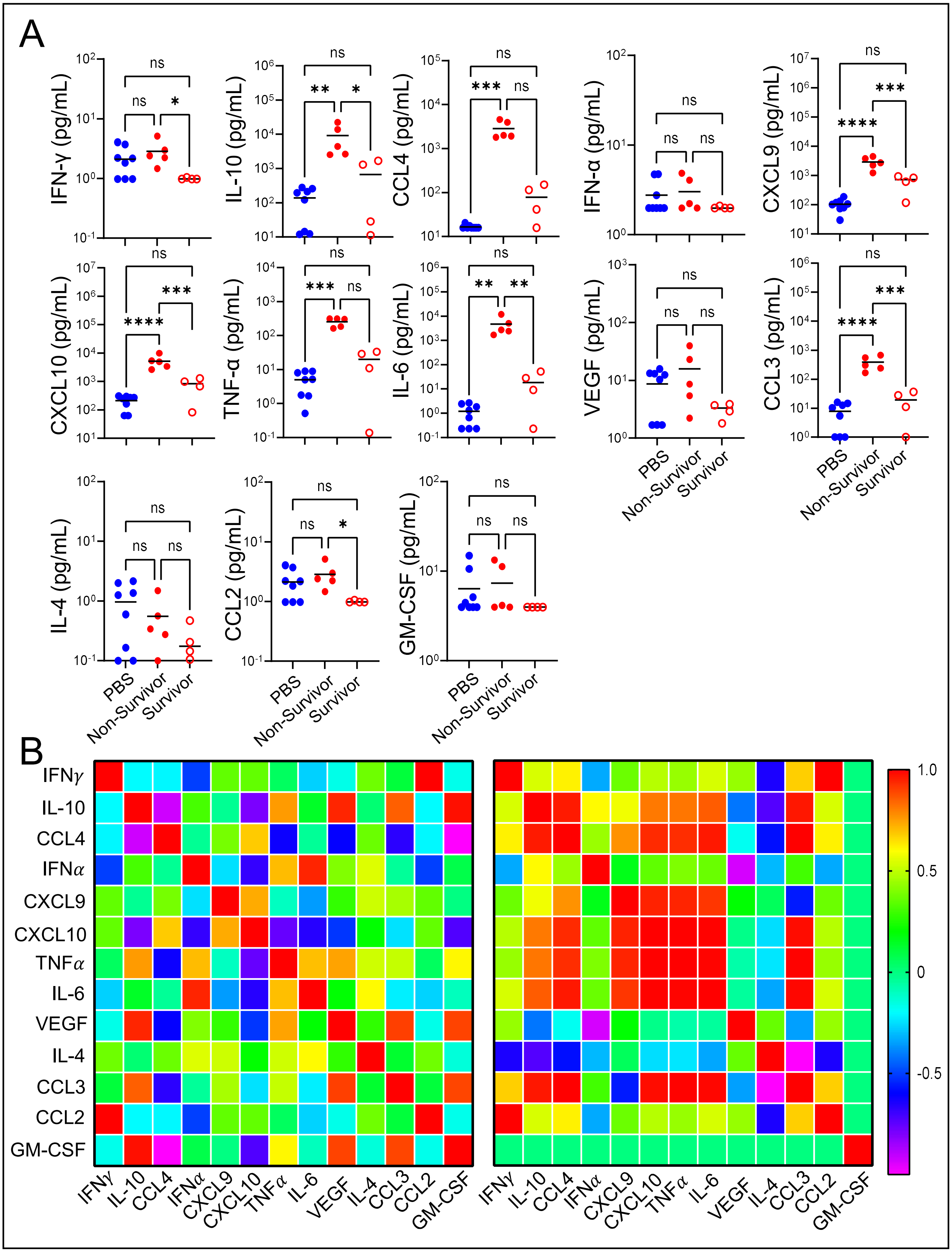

Supplementary Fig S10. (A) Pairwise comparisons of cytokine data between survivors and non-survivor mice 21hrs post sepsis induction. (B) Heatmap correlations of cytokines in non-survivors (left) and survivors (right) at 21 hrs post sepsis induction. Uncorrected Fisher's LSD or Kruskal-Wallis test; ns=not significant ($p>0.05$), *=$p<0.05$, **=$p<0.01$, ***=$p<0.001$.

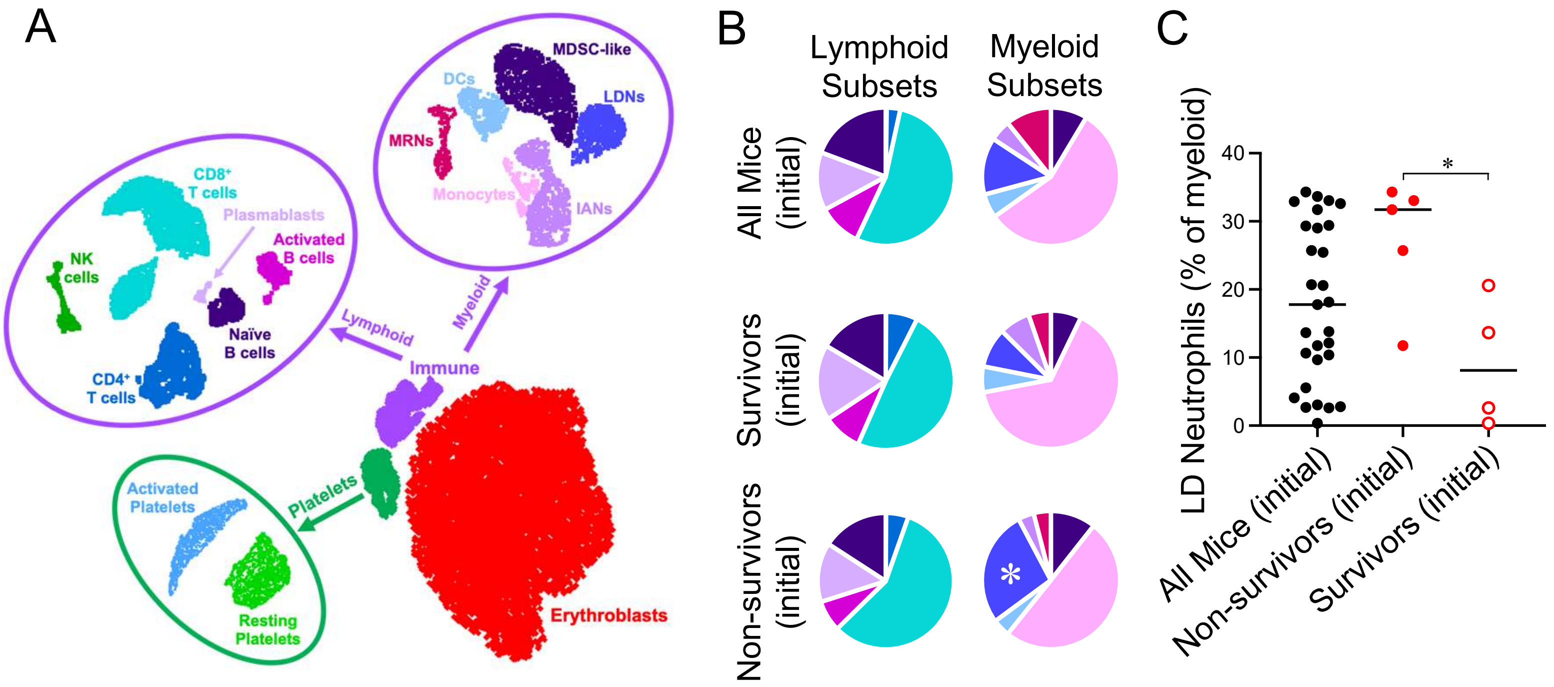

Supplementary Fig S11. Mice that go on to succumb to sepsis have higher initial levels of LD neutrophils. (A) Representative schematic of blood cell subsets analyzed by high dimensional flow cytometry. **(B)** Pie charts reflect relative abundance of subsets out of the total lymphoid or myeloid cell populations in mice <u>24hrs prior to sepsis induction</u>. Slice colors correspond to subset colors shown in A. **(C)** Variability among mice in levels of LD neutrophils <u>prior to sepsis induction</u> is depicted by black dots. The relative abundance of LD neutrophils in mice that would go on to succumb or survive sepsis are depicted by red dots and red-bordered white dots, respectively. Student's t-test; *=p<0.05.
